# Supplementary material for: Kidney intercalated cells are phagocytic and acidify internalized uropathogenic Escherichia coli
Source: Nat Commun. 2021 Apr 23;12:2405. doi: 10.1038/s41467-021-22672-5 (PMC8065053; doi:10.1038/s41467-021-22672-5)
Supplement: Supplementary file 1 — Supplementary Information [file 41467_2021_22672_MOESM1_ESM.pdf]

Supplementary Figure 1: The enrichment of ICs from human kidney samples

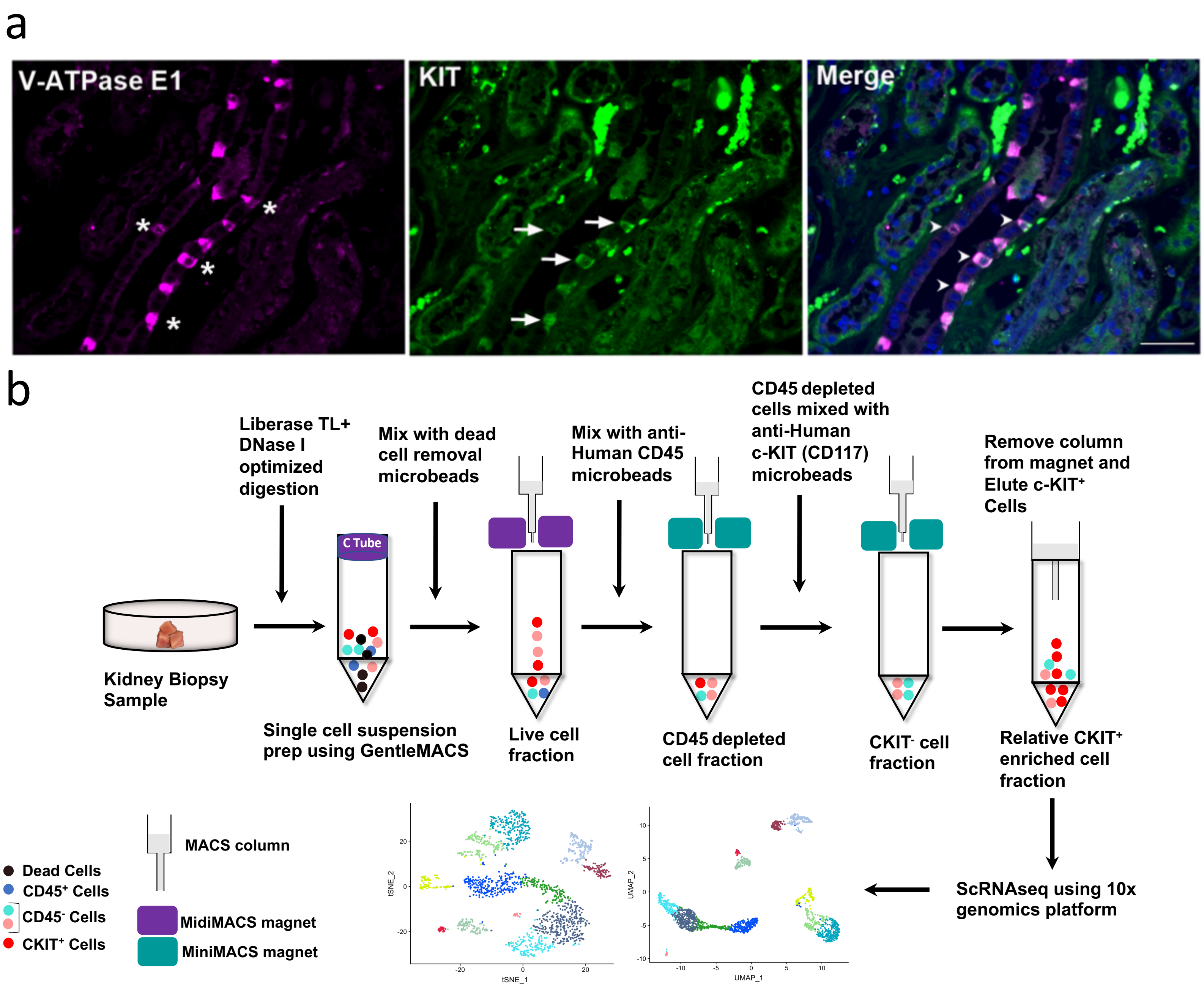

(a) E1 subunit of V-ATPase (magenta pseudo color from red) (asterisks, left panel) and cKIT (green, arrow, middle panel) are presented. C-KIT and the VATPase (E1 subunit) colocalized to the same cells (arrowheads, right panel) indicating that c-KIT represent a cell surface protein to target ICs for enrichment. Of note, c-KIT also stains stromal cells/telocytes and can intermittently stains some cells not positive for V-ATPase (E1 subunit) (green only cells, right panel) likely representing proximal tubules and loop of Henle cells seen in scRNAseq (Figure 1). These aforementioned cell types may represent the c-KIT staining that did not localize with the V-ATPase E1 subunits. Additionally, some green autofluorescence by red blood cells during IF imaging can occur. (b) Schematic representation of the removal of CD45+ cells and dead cells followed by magnetic enrichment of the remaining c-KIT positive cells. The images are representative of immunofluorescent results kidney sections from 2 distinct patients. Source data are provided as a Source Data File

Supplementary Figure 2: Confirmation of human IC enrichment in cells sorted using c-KIT microbeads

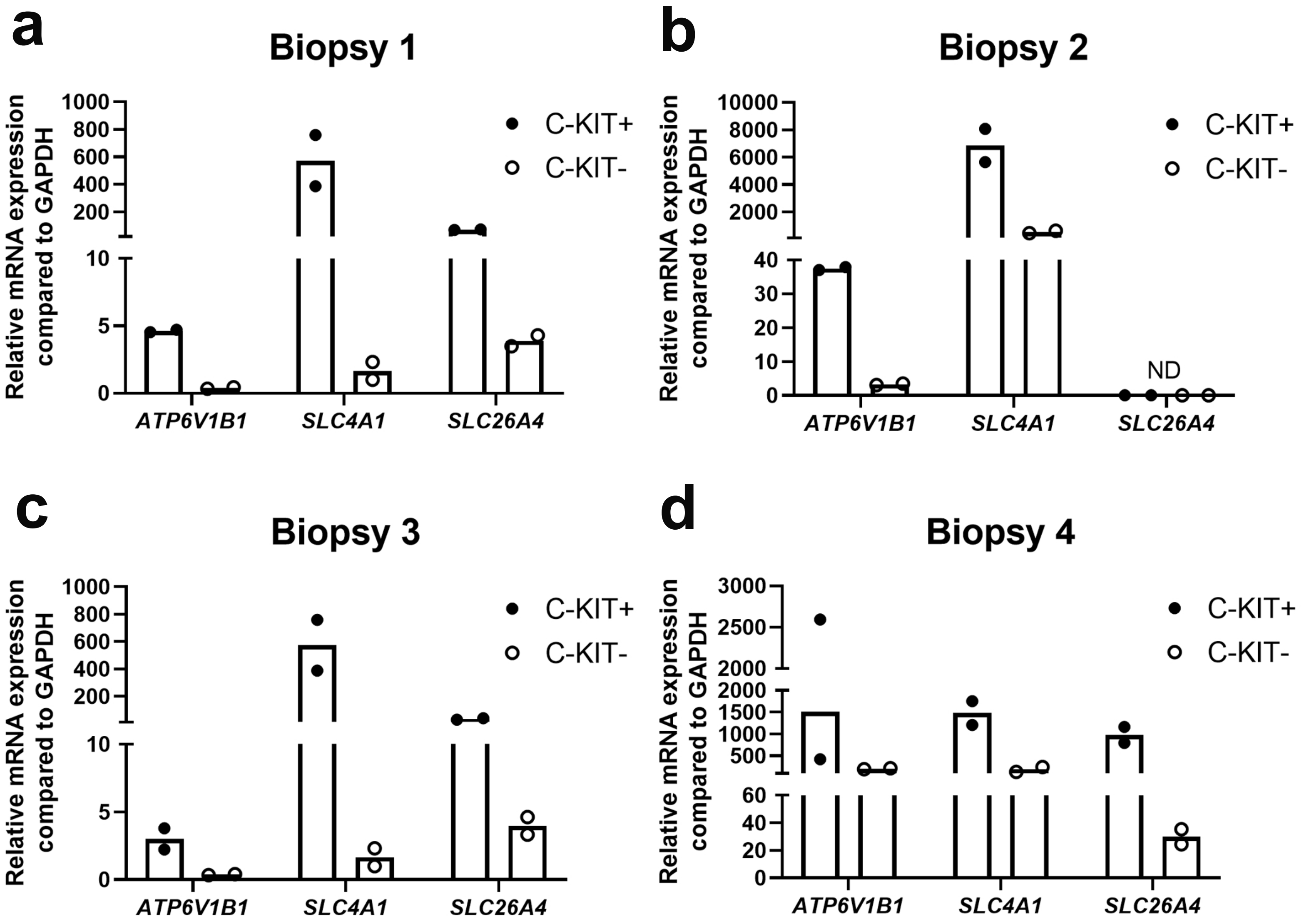

To confirm the overall IC enrichment when c-KIT positive kidney cells are magnetic sorted, normal margins from mass resection samples from 4 distinct individuals (a-d) were tested and scatter plots with superimposed bar graphs are presented. Each dot represents a run in a duplicate well. *ATP6V1B1 (IC marker), SLC4A1 (A-IC marker)* and *SLC26A4 (B-IC marker)* mRNA expression was measured by RT-PCR. The c-KIT enriched IC fraction was compared to the c-KIT negative fraction. We had variable degrees of enrichment for ICs in general (*ATPV1B1*), A-ICs (*SLC4A1*) and B-ICs (*SLC26A4*). *SLC26A4* was not detected in biopsy 2 (b) wells. Relative enrichment is presented from 4 distinct patient kidney tissue samples. Source data are provided as a Source Data File.

Supplementary Figure 3: Conserved markers within human kidney cell cluster 0

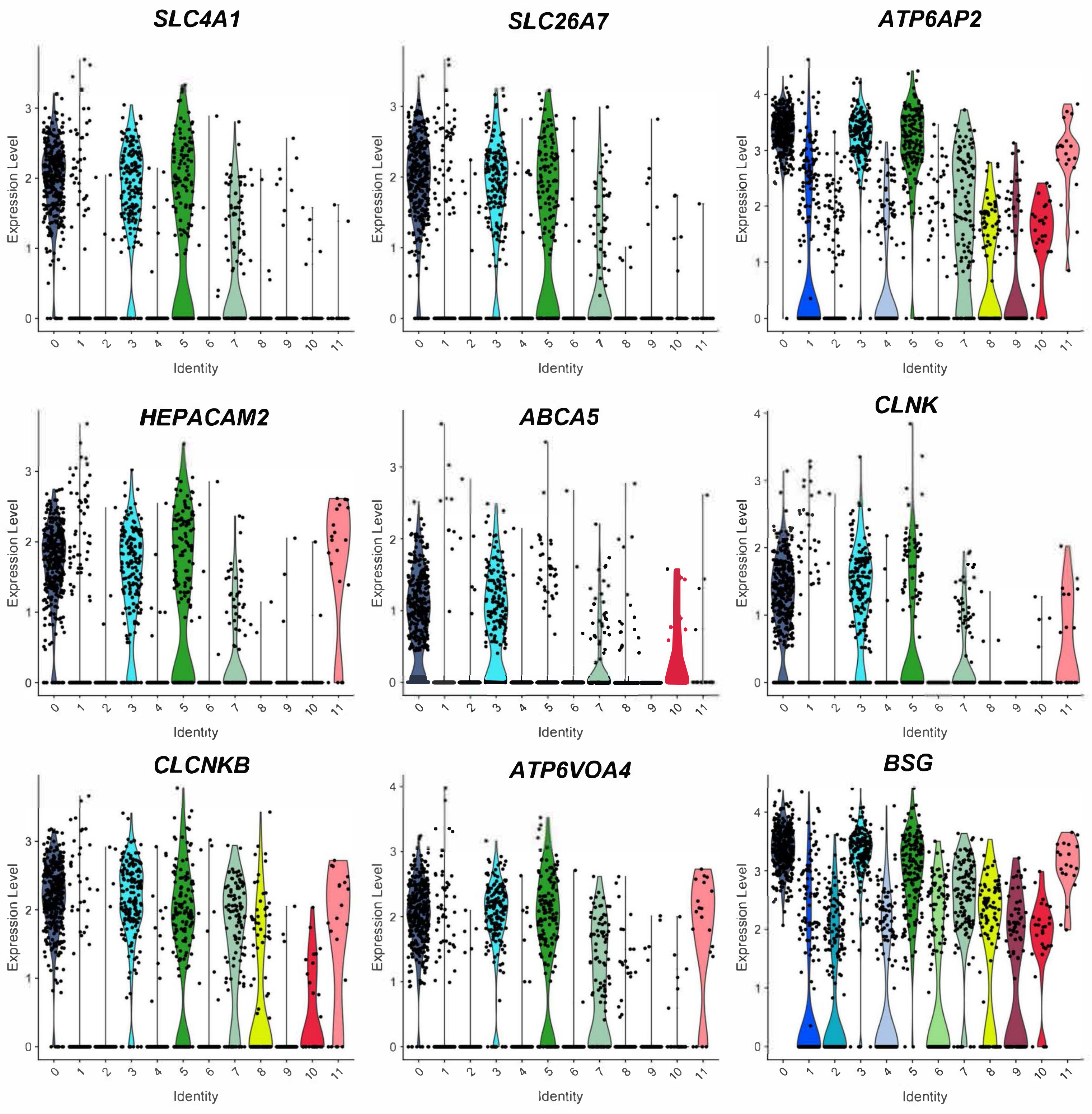

The 9 leading cell type markers identified on scRNAseq analysis for cluster 0 are presented. The data is presented with the relative expression on the y axis and cluster identity on the x axis. Violin plots are presented to demonstrate relative expression of the marker compared to other clusters. On the violin plots, each dot represents a single cell and the color is consistent with cluster color on the tSNE and UMAP plots (Figure 1a).

Supplementary Figure 4: Conserved markers within human kidney cell cluster 1

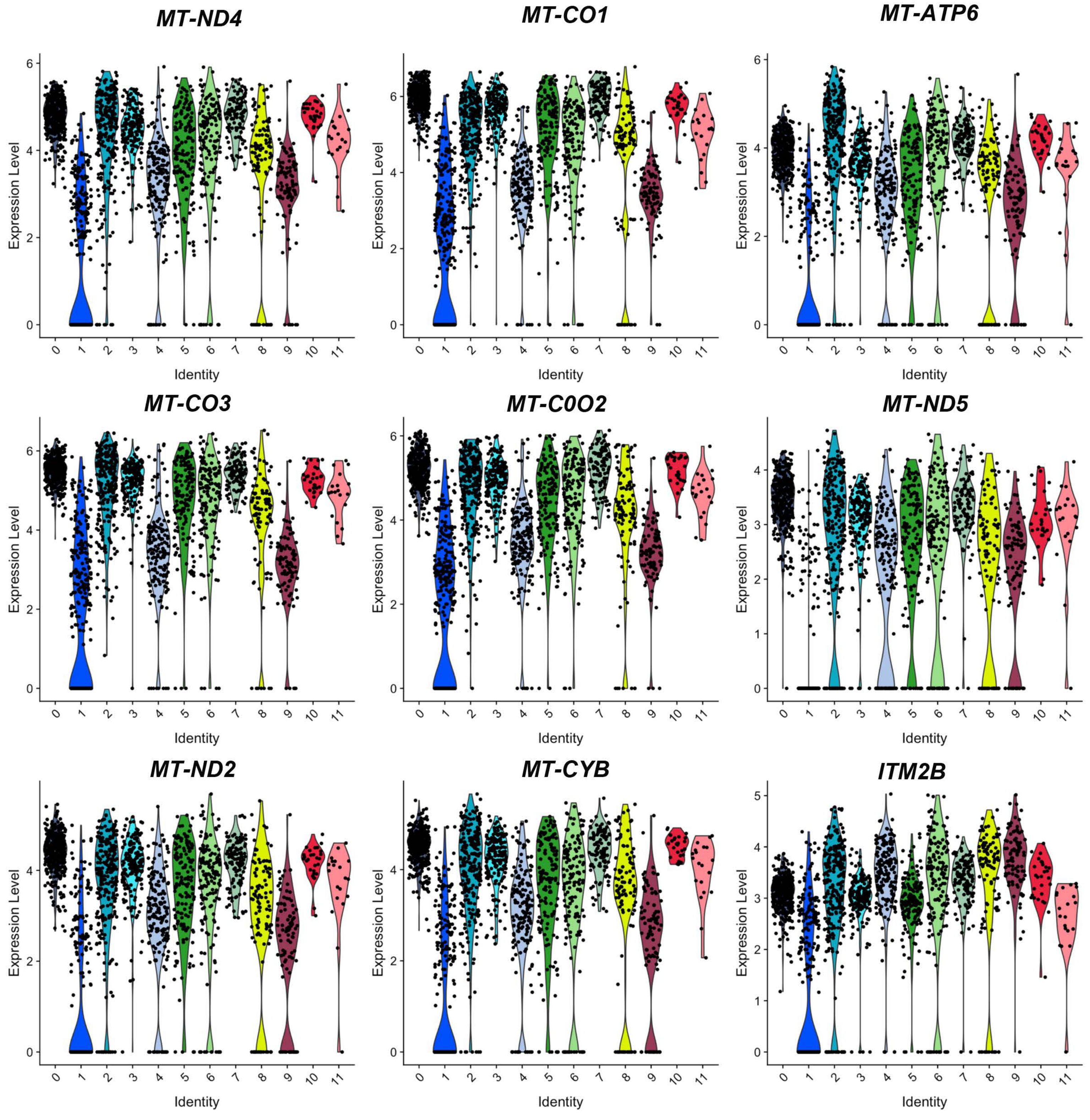

The 9 leading cell type markers identified on scRNAseq analysis for cluster 1 are presented. The data is presented with the relative expression on the y axis and cluster identity on the x axis. Violin plots are presented to demonstrate relative expression of the marker compared to other clusters. On the violin plots, each dot represents a single cell and the color is consistent with cluster color on the tSNE and UMAP plots (Figure 1a).

Supplementary Figure 5: Conserved markers within human kidney cell cluster 2

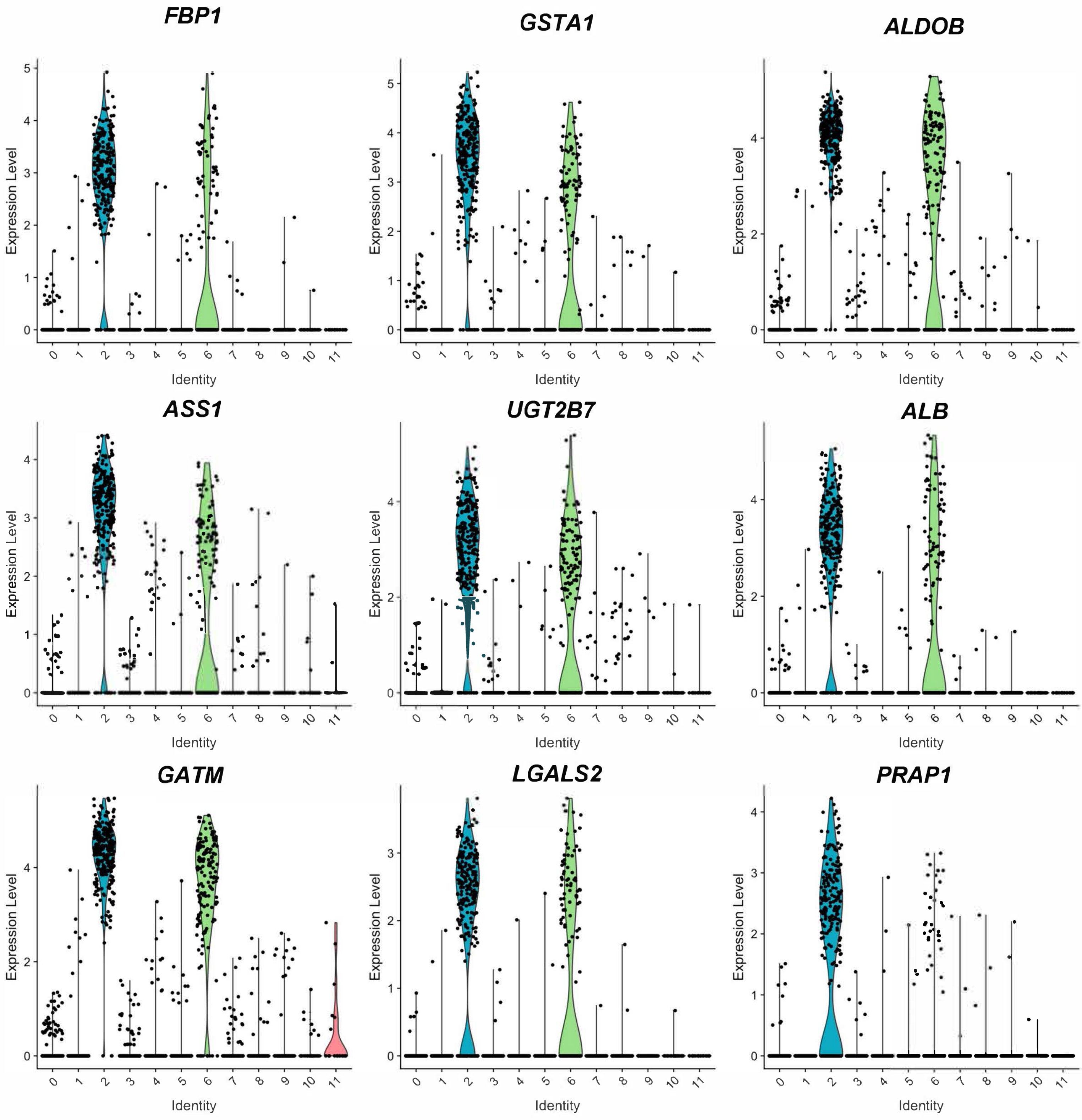

The 9 leading cell type markers identified on scRNAseq analysis for cluster 2 are presented. The data is presented with the relative expression on the y axis and cluster identity on the x axis. Violin plots are presented to demonstrate relative expression of the marker compared to other clusters. On the violin plots, each dot represents a single cell and the color is consistent with cluster color on the tSNE and UMAP plots (Figure 1a).

Supplementary Figure 6: Conserved markers within human kidney cell cluster 3

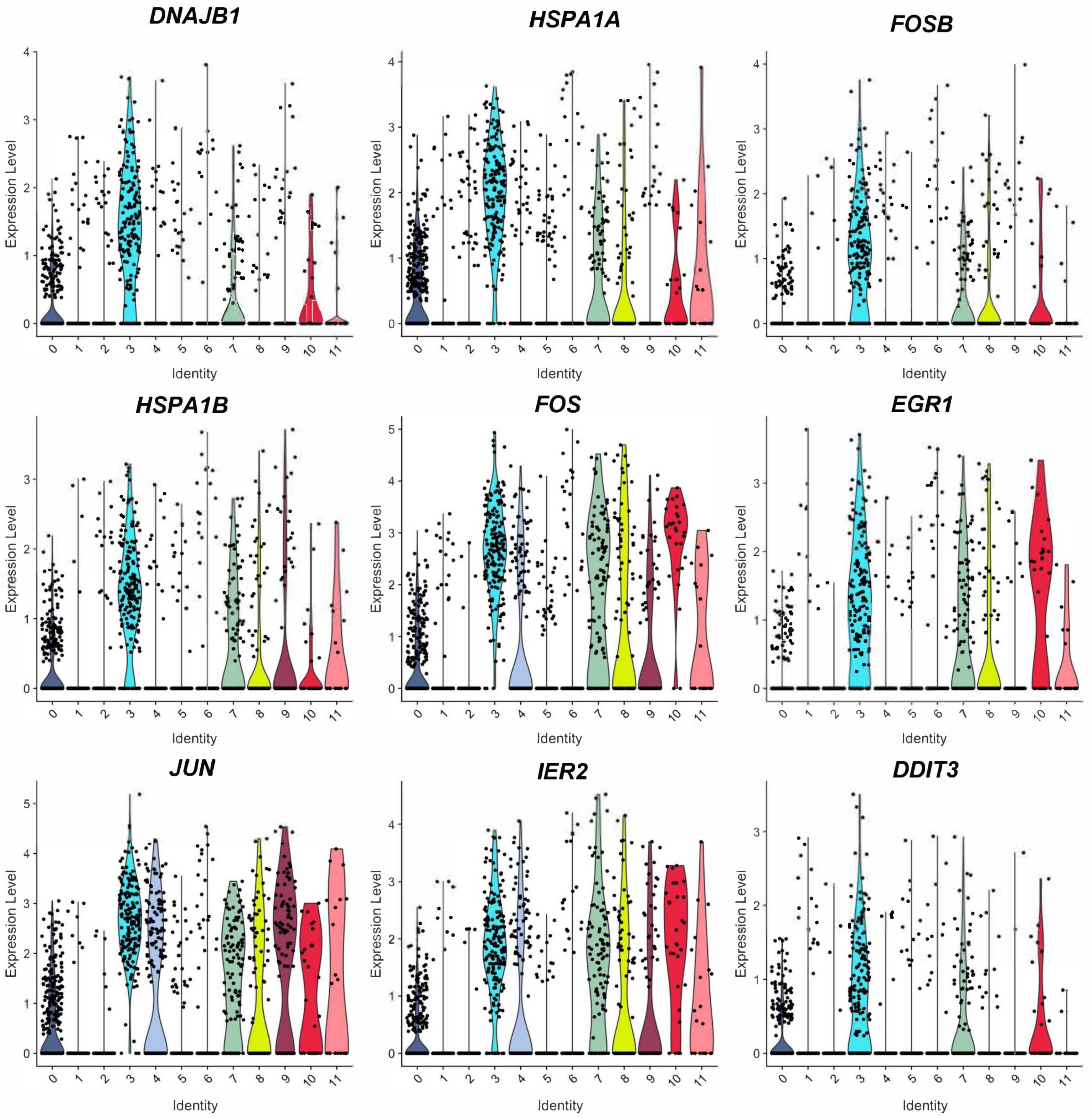

The 9 leading cell type markers identified on scRNAseq analysis for cluster 3 are presented. The data is presented with the relative expression on the y axis and cluster identity on the x axis. Violin plots are presented to demonstrate relative expression of the marker compared to other clusters. On the violin plots, each dot represents a single cell and the color is consistent with cluster color on the tSNE and UMAP plots (Figure 1a).

Supplementary Figure 7: Conserved markers within human kidney cell cluster 4

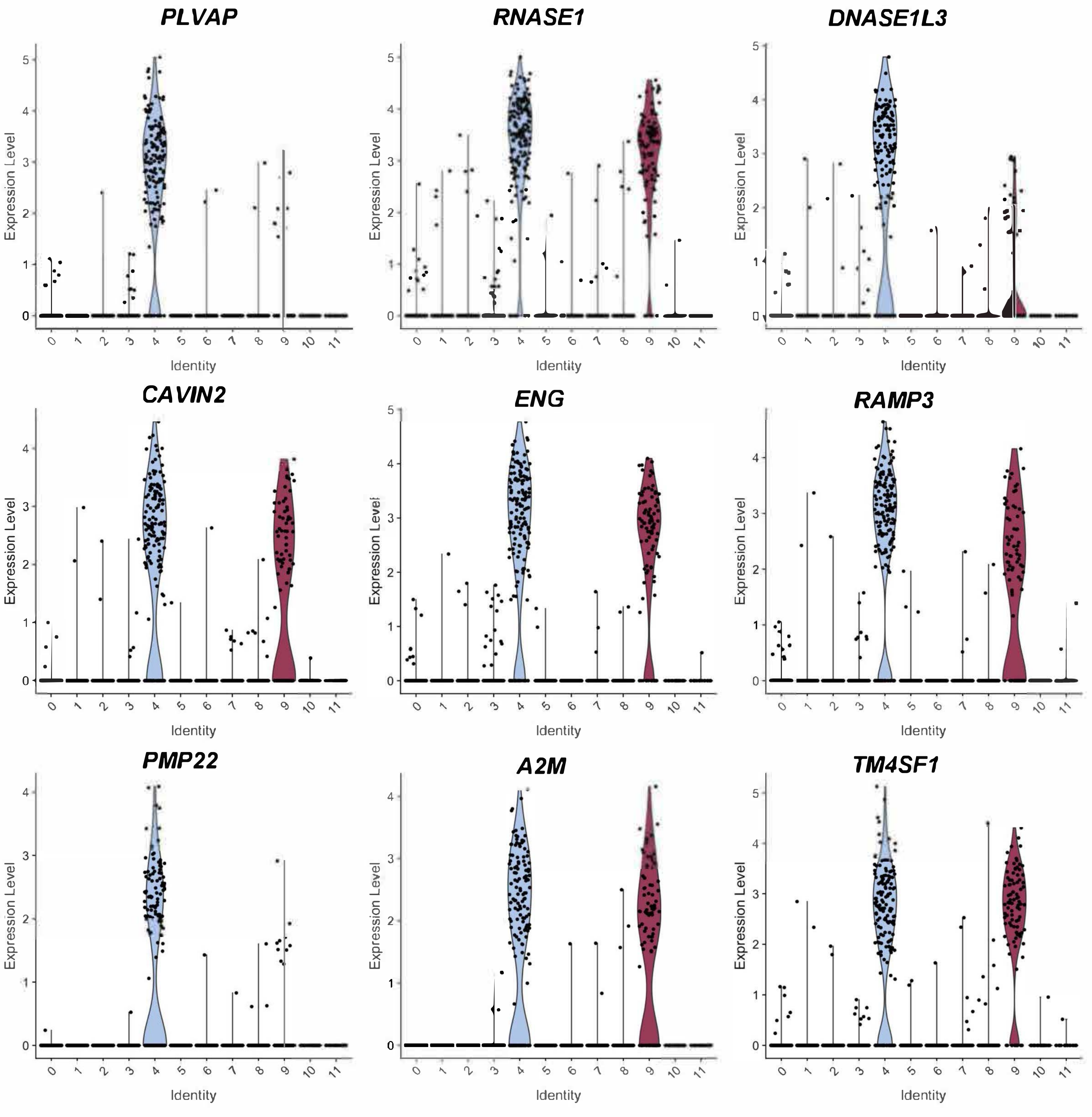

The 9 leading cell type markers identified on scRNAseq analysis for cluster 4 are presented. The data is presented with the relative expression on the y axis and cluster identity on the x axis. Violin plots are presented to demonstrate relative expression of the marker compared to other clusters. On the violin plots, each dot represents a single cell and the color is consistent with cluster color on the tSNE and UMAP plots (Figure 1a).

Supplementary Figure 8: Conserved markers within human kidney cell cluster 5

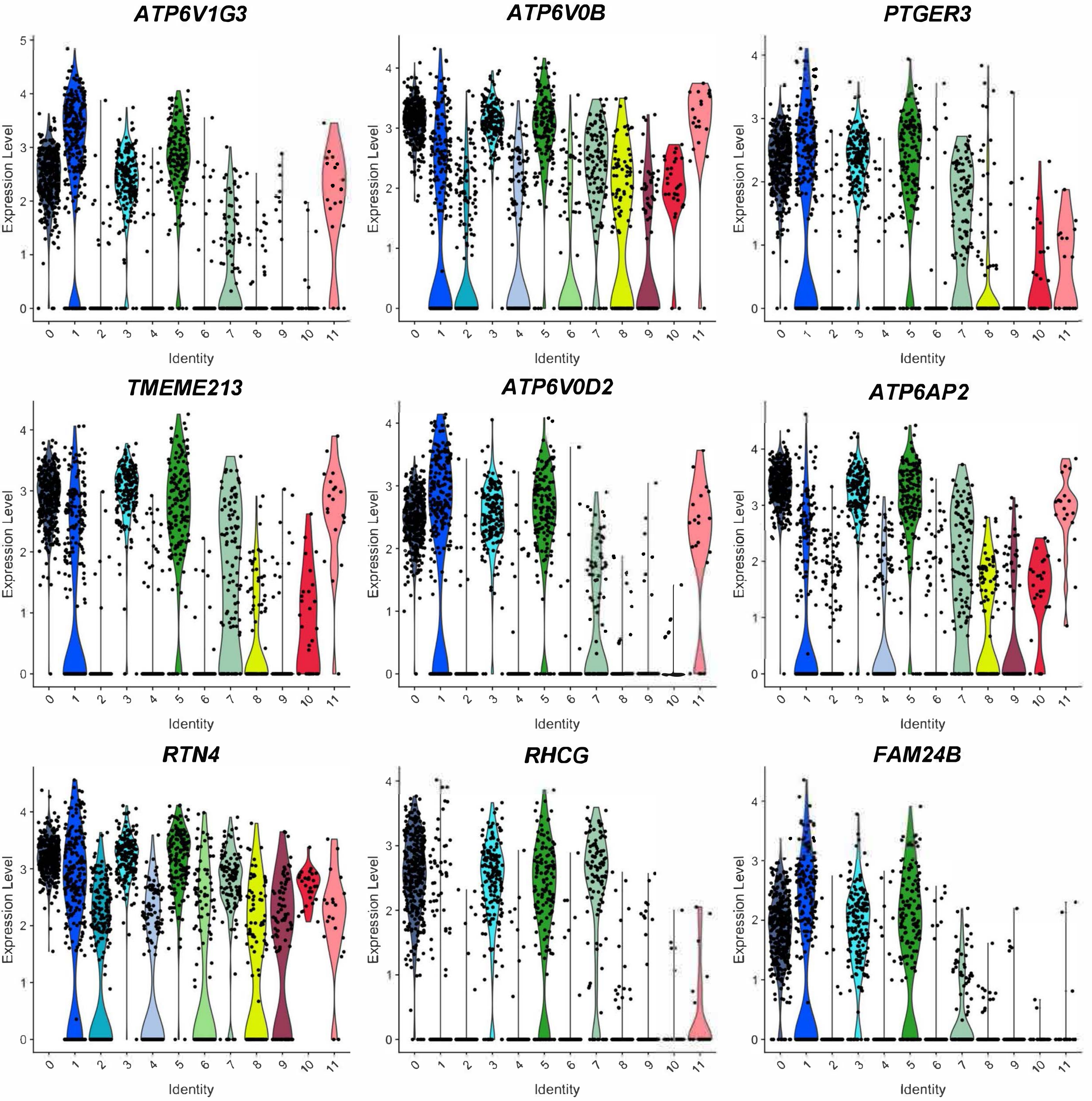

The 9 leading cell type markers identified on scRNAseq analysis for cluster 5 are presented. The data is presented with the relative expression on the y axis and cluster identity on the x axis. Violin plots are presented to demonstrate relative expression of the marker compared to other clusters. On the violin plots, each dot represents a single cell and the color is consistent with cluster color on the tSNE and UMAP plots (Figure 1a).

Supplementary Figure 9: Conserved markers within human kidney cell cluster 6

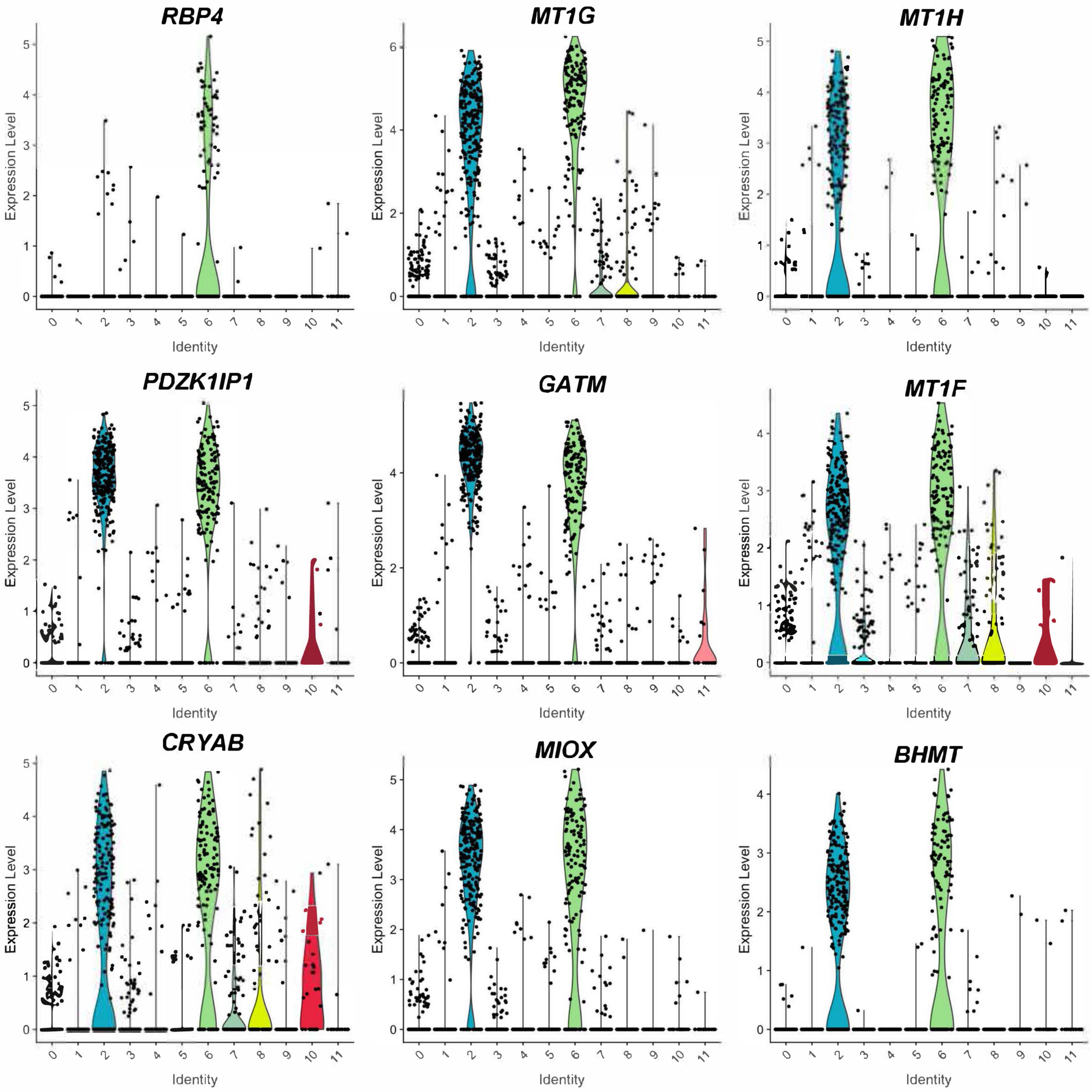

The 9 leading cell type markers identified on scRNAseq analysis for cluster 6 are presented. The data is presented with the relative expression on the y axis and cluster identity on the x axis. Violin plots are presented to demonstrate relative expression of the marker compared to other clusters. On the violin plots, each dot represents a single cell and the color is consistent with cluster color on the tSNE and UMAP plots (Figure 1a).

Supplementary Figure 10: Conserved markers within human kidney cell cluster 7

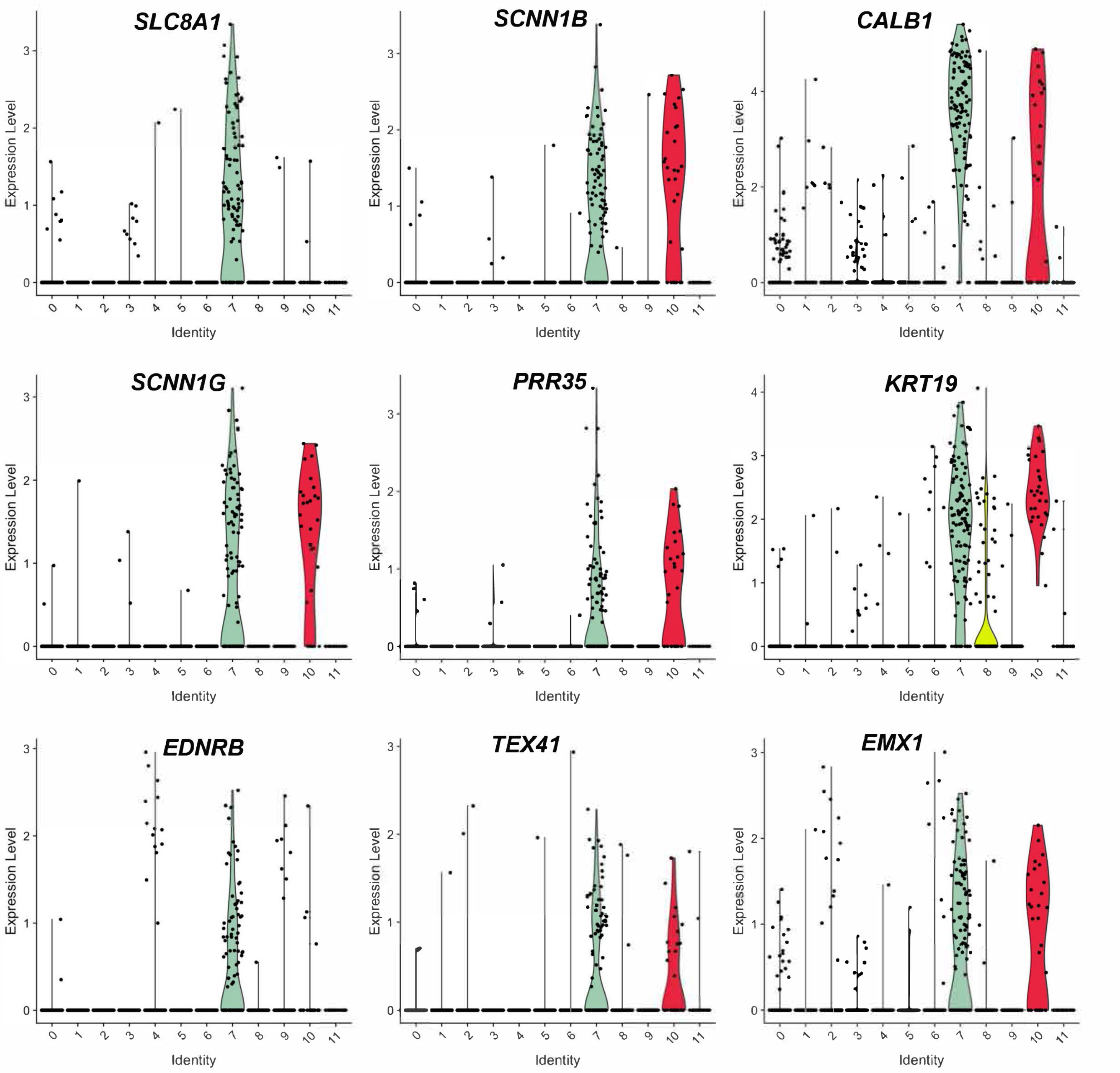

The 9 leading cell type markers identified on scRNAseq analysis for cluster 7 are presented. The data is presented with the relative expression on the y axis and cluster identity on the x axis. Violin plots are presented to demonstrate relative expression of the marker compared to other clusters. On the violin plots, each dot represents a single cell and the color is consistent with cluster color on the tSNE and UMAP plots (Figure 1a).

Supplementary Figure 11: Conserved markers within human kidney cell cluster 8

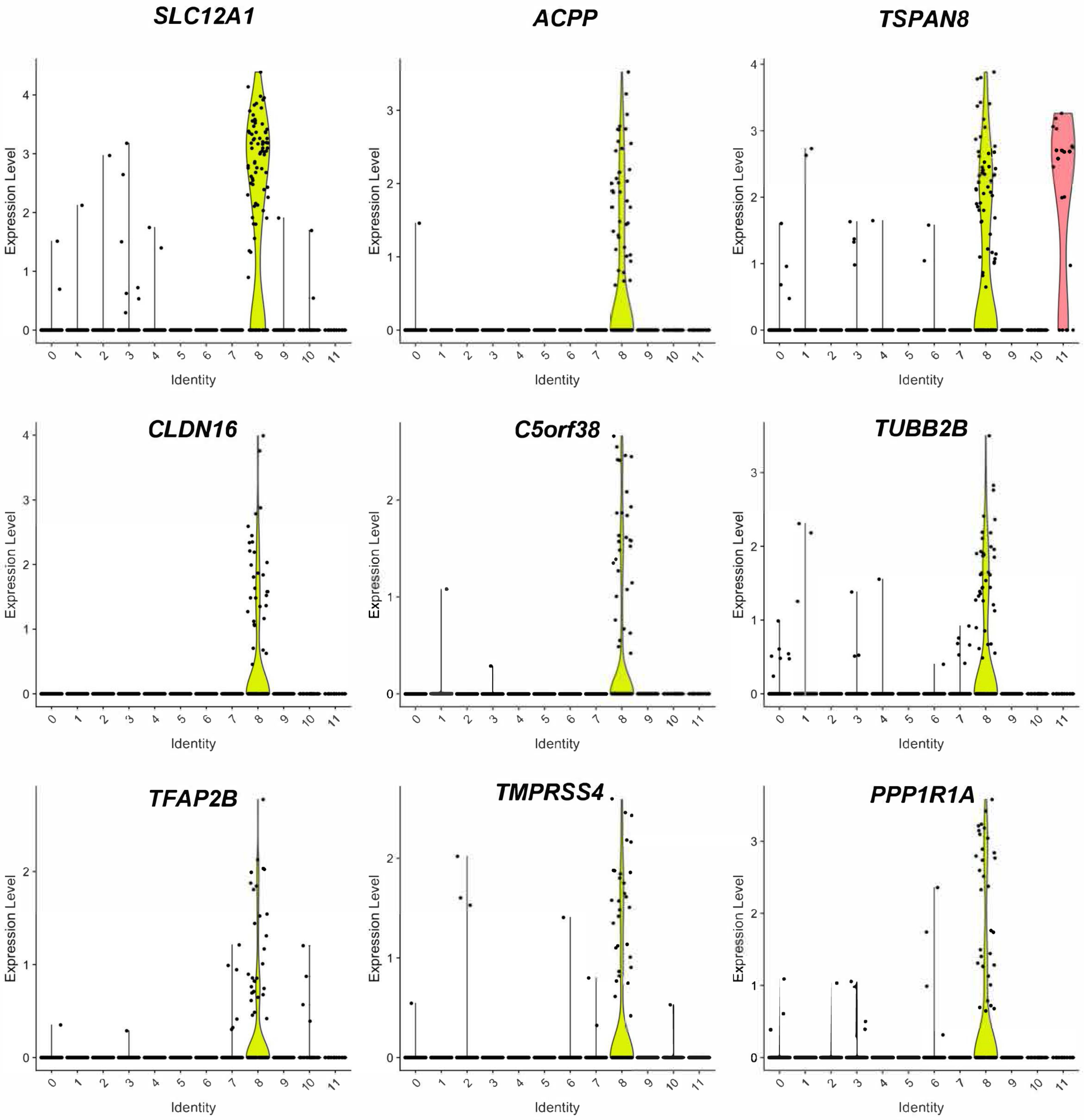

The 9 leading cell type markers identified on scRNAseq analysis for cluster 8 are presented. The data is presented with the relative expression on the y axis and cluster identity on the x axis. Violin plots are presented to demonstrate relative expression of the marker compared to other clusters. On the violin plots, each dot represents a single cell and the color is consistent with cluster color on the tSNE and UMAP plots (Figure 1a).

Supplementary Figure 12: Conserved markers within human kidney cell cluster 9

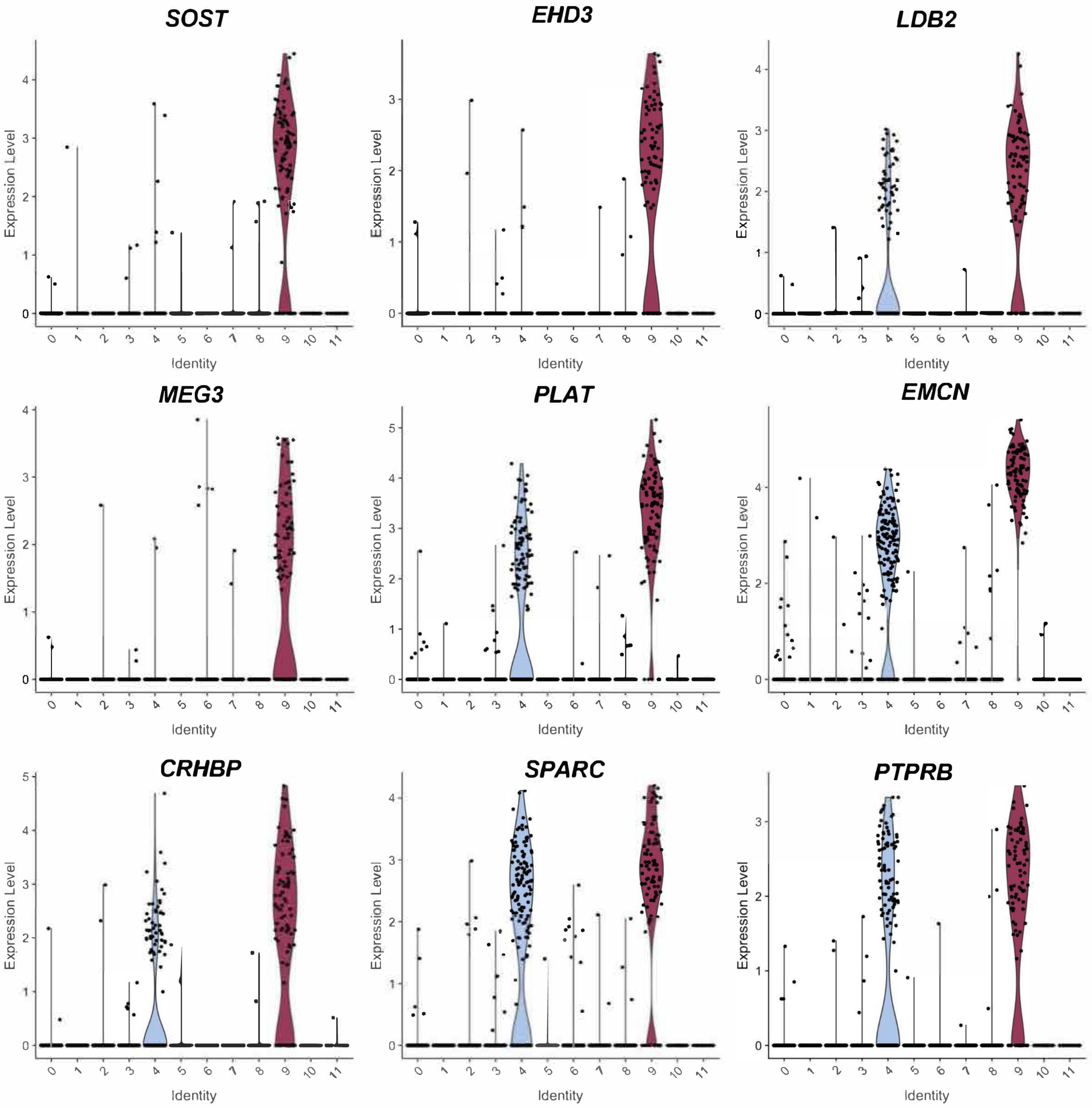

The 9 leading cell type markers identified on scRNAseq analysis for cluster 9 are presented. The data is presented with the relative expression on the y axis and cluster identity on the x axis. Violin plots are presented to demonstrate relative expression of the marker compared to other clusters. On the violin plots, each dot represents a single cell and the color is consistent with cluster color on the tSNE and UMAP plots (Figure 1a).

Supplementary Figure 13: Conserved markers within human kidney cell cluster 10

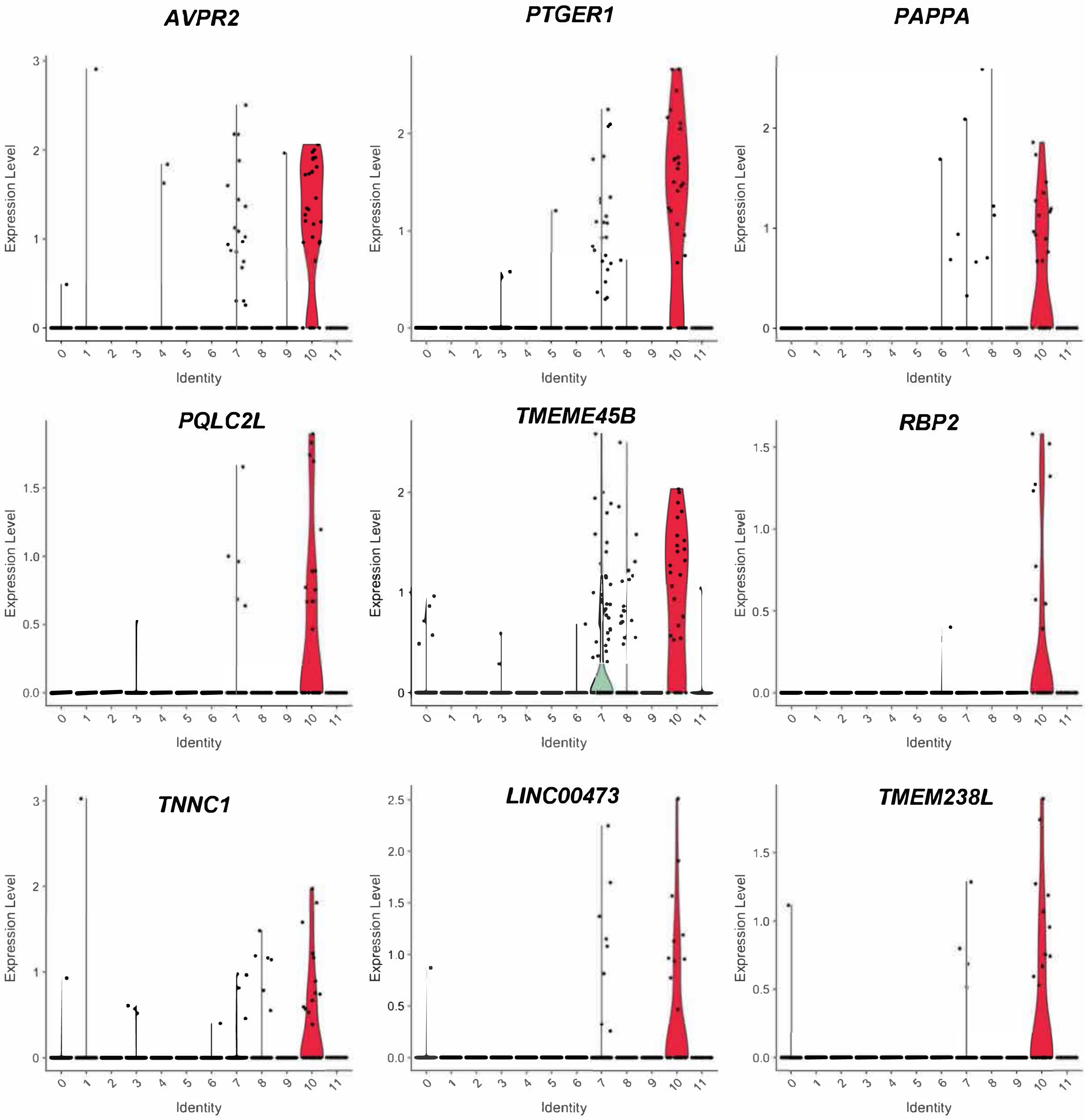

The 9 leading cell type markers identified on scRNAseq analysis for cluster 10 are presented. The data is presented with the relative expression on the y axis and cluster identity on the x axis. Violin plots are presented to demonstrate relative expression of the marker compared to other clusters. On the violin plots, each dot represents a single cell and the color is consistent with cluster color on the tSNE and UMAP plots (Figure 1a).

Supplementary Figure 14: Conserved markers within human kidney cell cluster 11

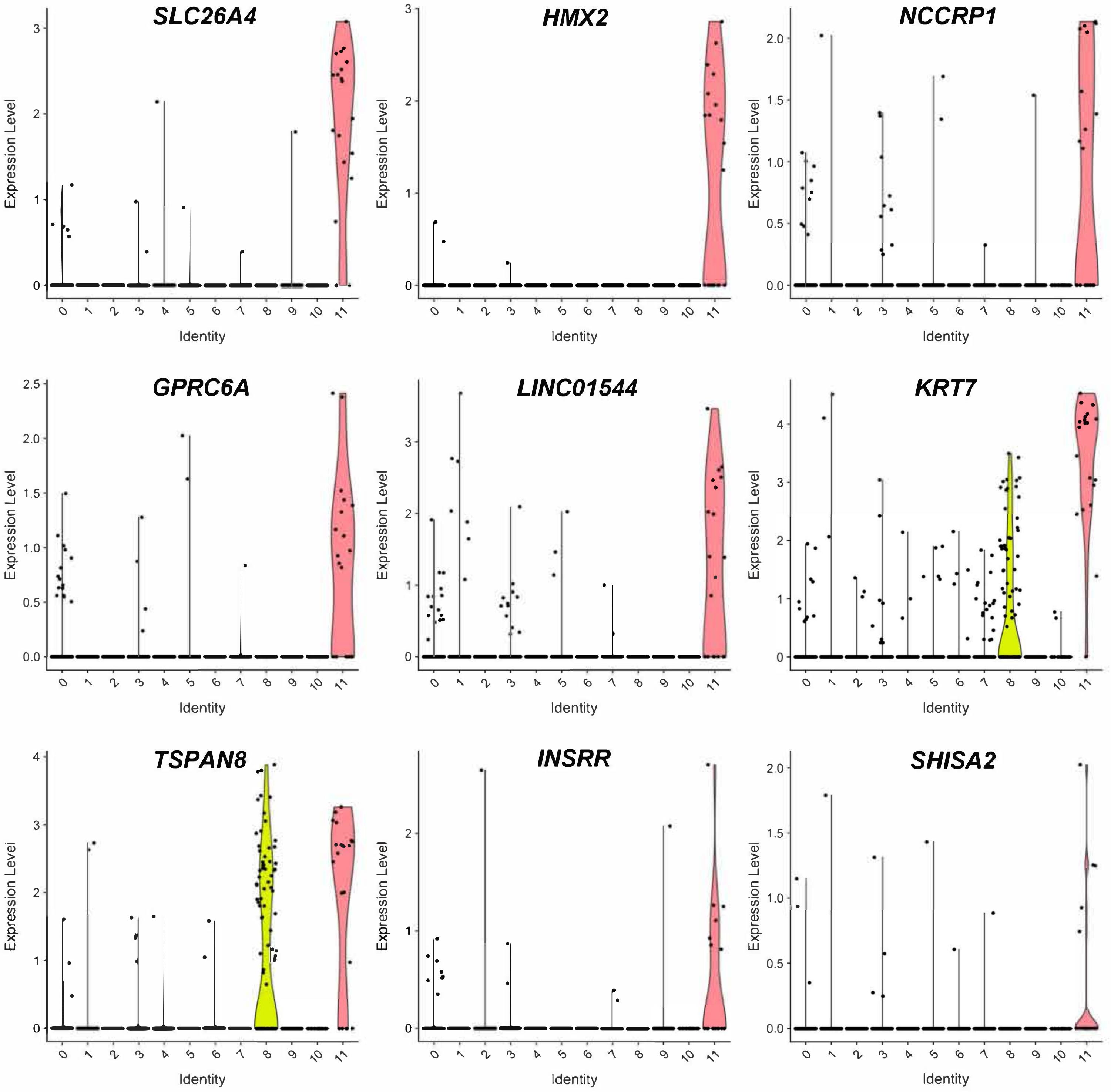

The 9 leading cell type markers identified on scRNAseq analysis for cluster 11 are presented. The data is presented with the relative expression on the y axis and cluster identity on the x axis. Violin plots are presented to demonstrate relative expression of the marker compared to other clusters. On the violin plots, each dot represents a single cell and the color is consistent with cluster color on the tSNE and UMAP plots (Figure 1a).

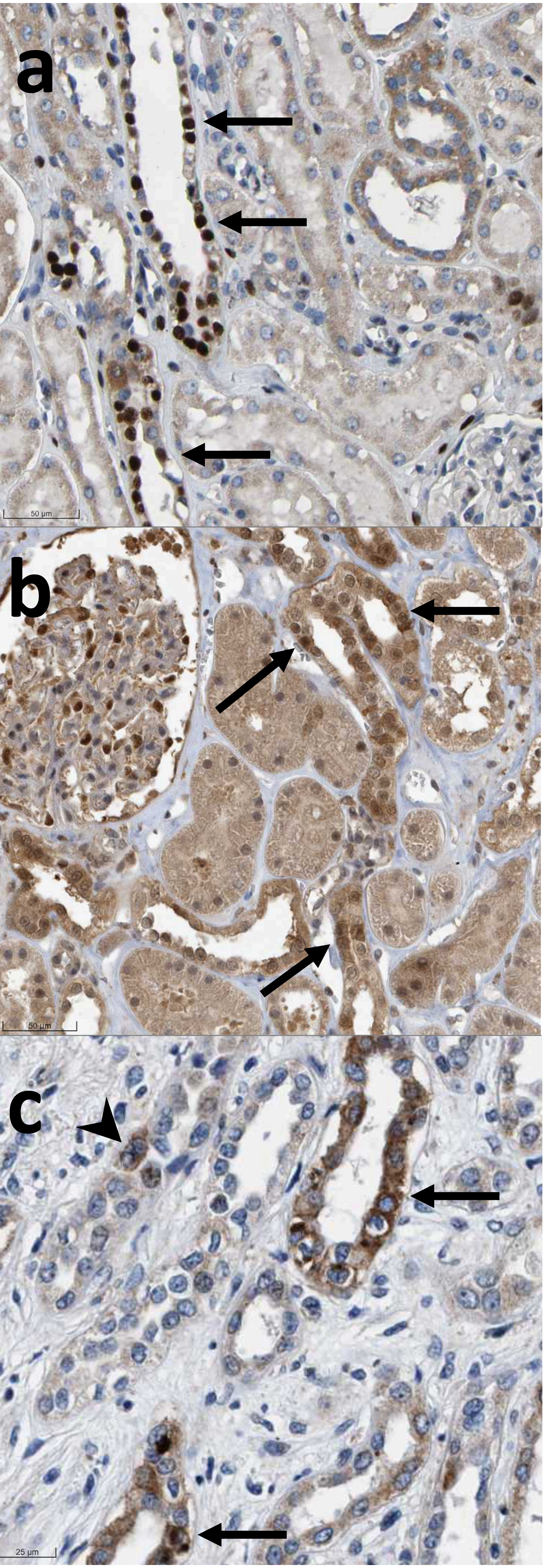

Supplementary Figure 15: EGR1, HSPA1A and SLC8A1 protein expression in normal kidney tissue

Immunohistochemistry is presented where positive immunolabeling is noted by brown staining. Kidney protein expression of EGR1 (a), HSPA1A (b) and SLC8A1 (c) in normal human tissue from the Human Protein Atlas version 20.0 [v20.0.proteinatlas.org] obtained from a 16-year-old male, 41-year- old female and 59-year-old male respectively (a) EGR1 immunostains the nuclei of some, but not all isolated tubules (arrows). (b) HSPA1A has more pronounced immunolabeling in select isolated tubules (arrows), the HSPA2A immunolabeling is heterogenous and mostly cytoplasmic, but some nuclei are lightly immunostained as well. (c) SLC8A1 consistently immunostains cells in occasional tubules (arrows) while other tubules only had rare isolated tubules (arrowheads). The images presented include 1 individual for each protein and are representative from 3 individuals presented in the Human Protein Atlas for EGR1, 2 for HSPA1A and 3 for SLC8A1. Image credit: Human Protein Atlas.

(a) Tissue ID 1767, antibody CAB019427, [https://www.proteinatlas.org/ENSG00000120738-EGR1/tissue/kidney#img]
(b) Tissue ID 2530, antibody CAB017451, [https://www.proteinatlas.org/ENSG00000204389-HSPA1A/tissue/kidney#img])
(c) Tissue ID 3229, antibody CAB022694, [https://www.proteinatlas.org/ENSG00000183023-SLC8A1/tissue/kidney#img]

Supplementary Figure 16: Dot plots of select innate immune genes identified by scRNAseq

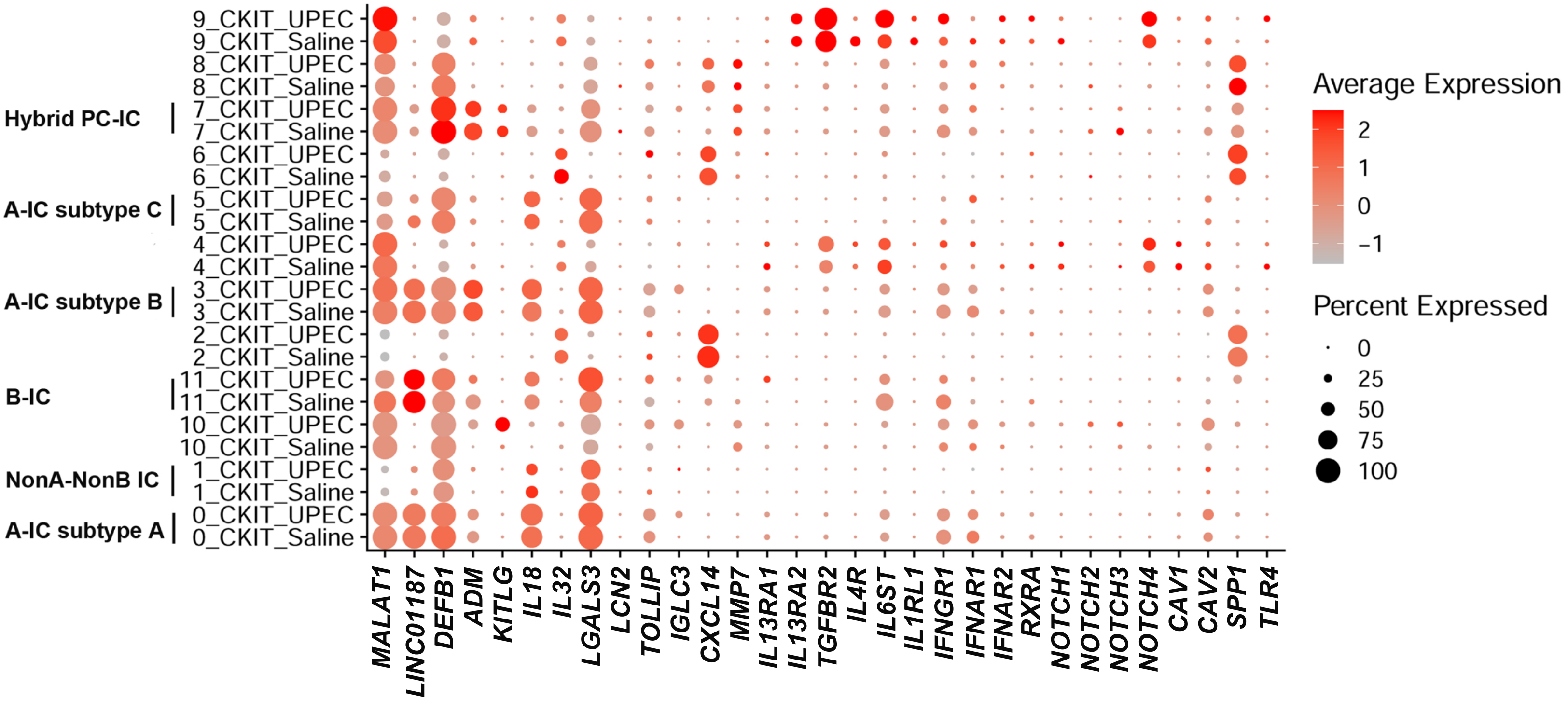

Dot plots to demonstrate the relative mRNA expression of innate immune genes that were identified or not identified in clusters is presented. The genes included in the innate immune profile are metastasis associated lung adenocarcinoma transcript 1 (*MALAT1*), long intergenic non-protein coding RNA 1184 (*LINC01184*), beta defensin 1 (*DEFB1*), adrenomedullin (*ADM*), KIT ligand (*KITLG*), interleukin 18 (*IL18*), interleukin 32 (*IL32*), galectin 3 (*LGALS3*), lipocalin 2 (*LCN2*), toll Interacting Protein (*TOLLIP)*, immunoglobulin lambda constant 3 (*IGLC3*), C-X-C motif chemokine ligand 14 (*CXCL14)*, matrix metallopeptidase 7 (*MMP7*), interleukin 13 receptor subunit alpha 2 (*IL13RA2*), transforming growth factor beta receptor 2 (*TGFBR2*), interleukin 4 receptor (IL4R), interleukin 6 signal transducer (*IL6ST*), interleukin 1 receptor like 1 (*IL1RL1*), interferon alpha and beta receptor subunit 1 and 2 (*IFNAR1* and *IFNAR2*), retinoid X receptor alpha (*RXRA*), notch receptors NOTCH 1, 2, 3 ,4 (*NOTCH1*, *NOTCH2*, *NOTCH3* and *NOTCH4*), Caveolin 1 and 2 (*CAV1* and *CAV2*), secreted phosphoprotein 1 (*SPP1*) and toll like receptor (*TLR4*).

Supplementary Figure 17: Volcano plots of select innate immune genes identified by scRNAseq to highlight differential expression between UPEC and saline exposed cells

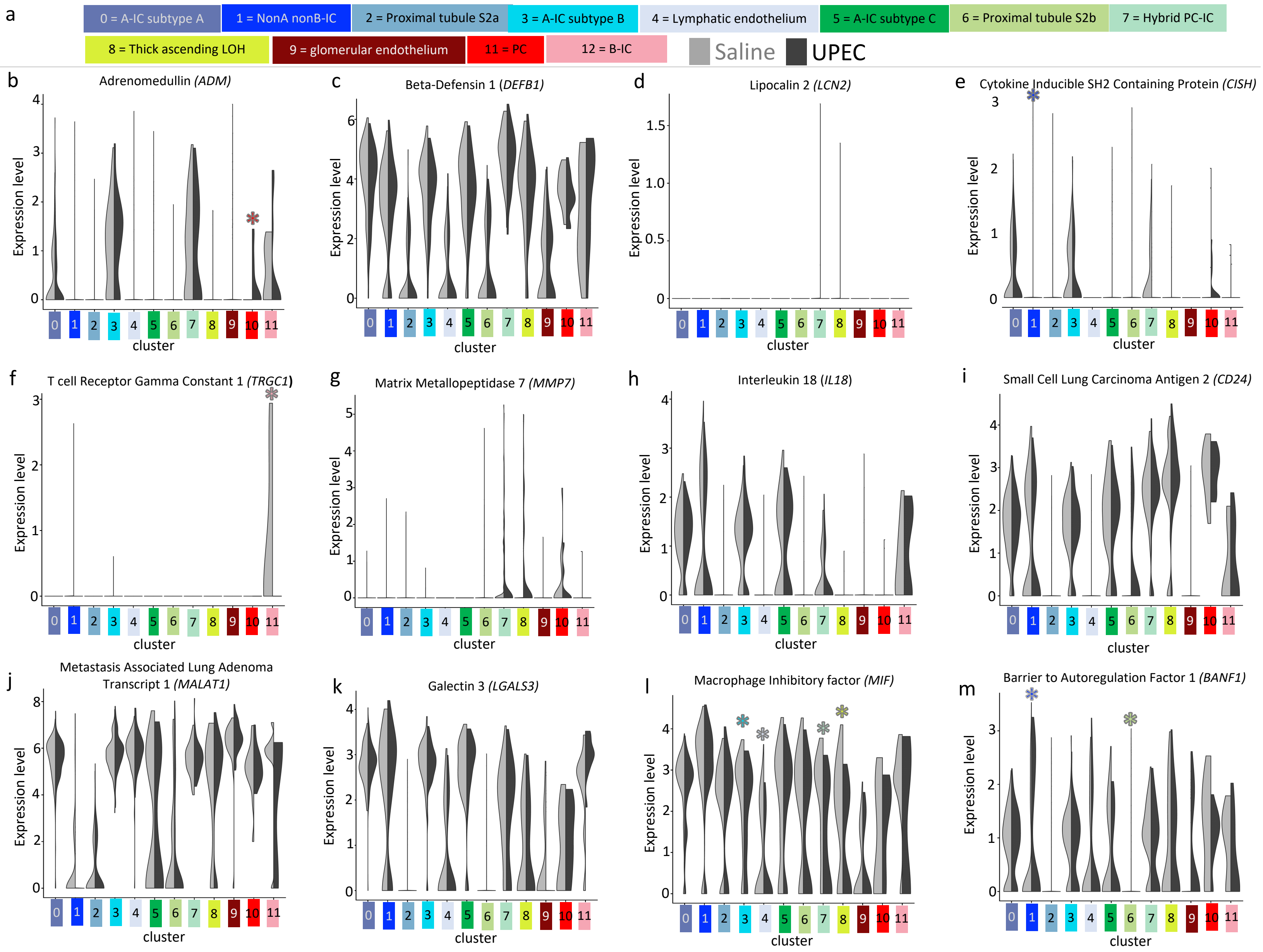

Asterisks indicate statistically significant differences between UPEC and saline exposed cells. (a) The color in the key correlates to cluster color in Figure 1a and the x axis. Included innate immune effectors include genes for antimicrobial peptides such as adrenomedullin (*ADM*) (b), inflammatory response mediators including cytokine inducible SH2 containing protein (*CISH*) (c), interleukin 18 (*IL18*) (d) and T cell receptor gamma constant 1 (*TRGC1*) (e). Additionally the nutrient metal scavenger lipocalin 2 (*LCN2*) (f), the antimicrobial peptide activator matrix metalloproteinase 7 (*MMP7*) (g), the stress response regulator barrier to autointegration factor (*BANF1*) (h), the antimicrobial peptide beta defensin 1 (*DEFB1*) (i), the pattern recognition receptor galectin 3 (*LGAL3*) (j) along with the innate immune response mediators macrophage migration inhibitory factor (*MIF*) (k) and metastasis associated lung adenocarcinoma transcript 1 (*MALAT12*) (l) along with signal transducer CD24 (*CD24*) (m) have their gene expression presented. The “Findmarkers” function of Seuret uses the Wilcoxon rank sum test to compare differences between groups.

Supplementary Figure 18: *RNASE7* expression is intermittently present in c-KIT+ (ICs) but not in c-KIT- (non-ICs)

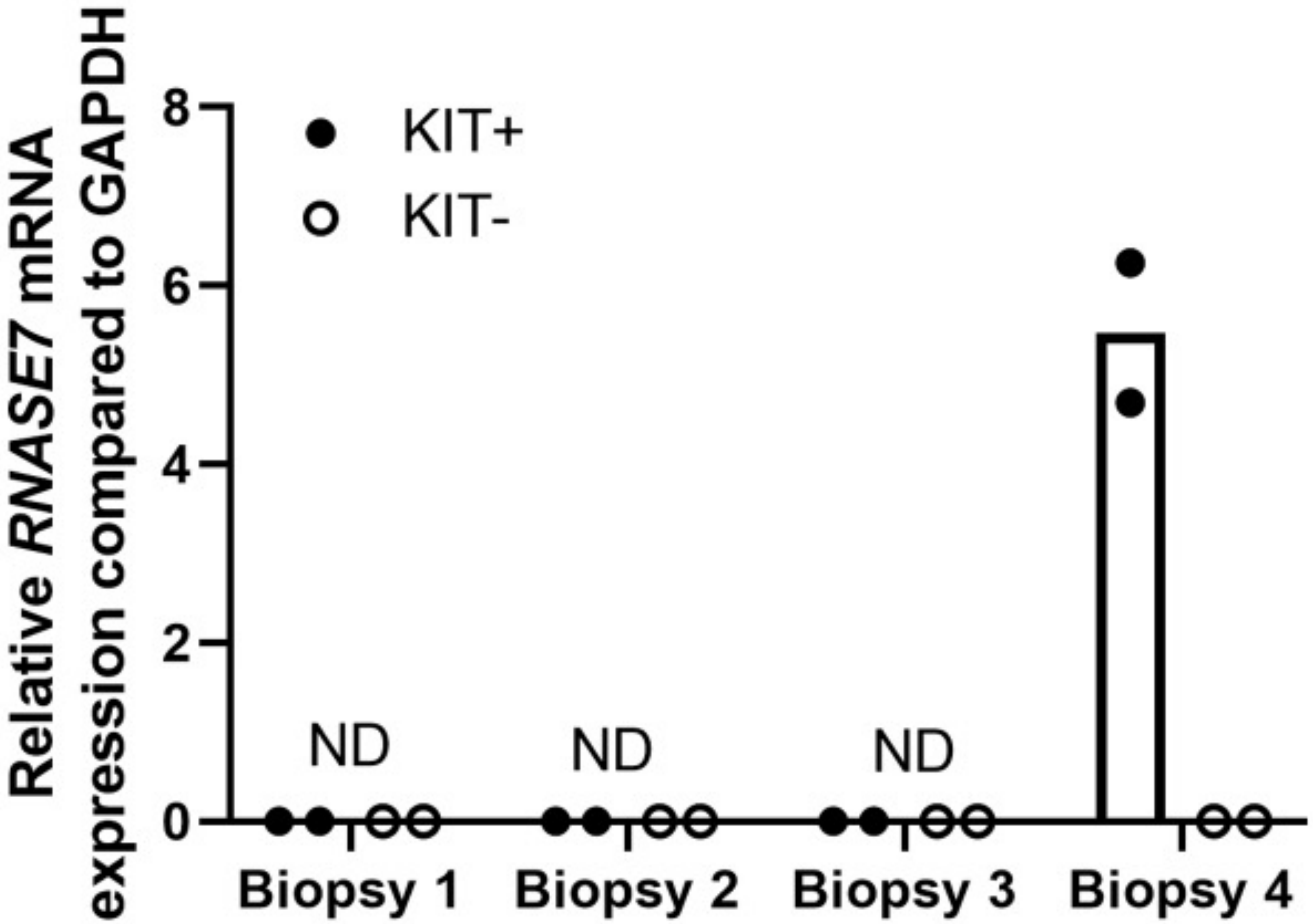

RNASE7 mRNA expression in ICs vs non-ICs magnetically enriched from normal margins following kidney mass resection. *RNASE7* was normalized to glyceraldehyde 3-phosphate dehydrogenase *(GAPDH)* for this comparison. Scatter plots with superimposed bar graphs are presented. *RNASE7* is only present in ICs from 1 patient (biopsy 4) and absent in non-ICs from all patients. Each dot represents a RT-PCR run in a duplicate well N= 4 kidney tissue from distinct individuals. Source data are provided as a Source Data File. ND = not detected.

Supplementary Figure 19: Some collecting cell types have no changes in leading biological pathways with saline vs UPEC exposure

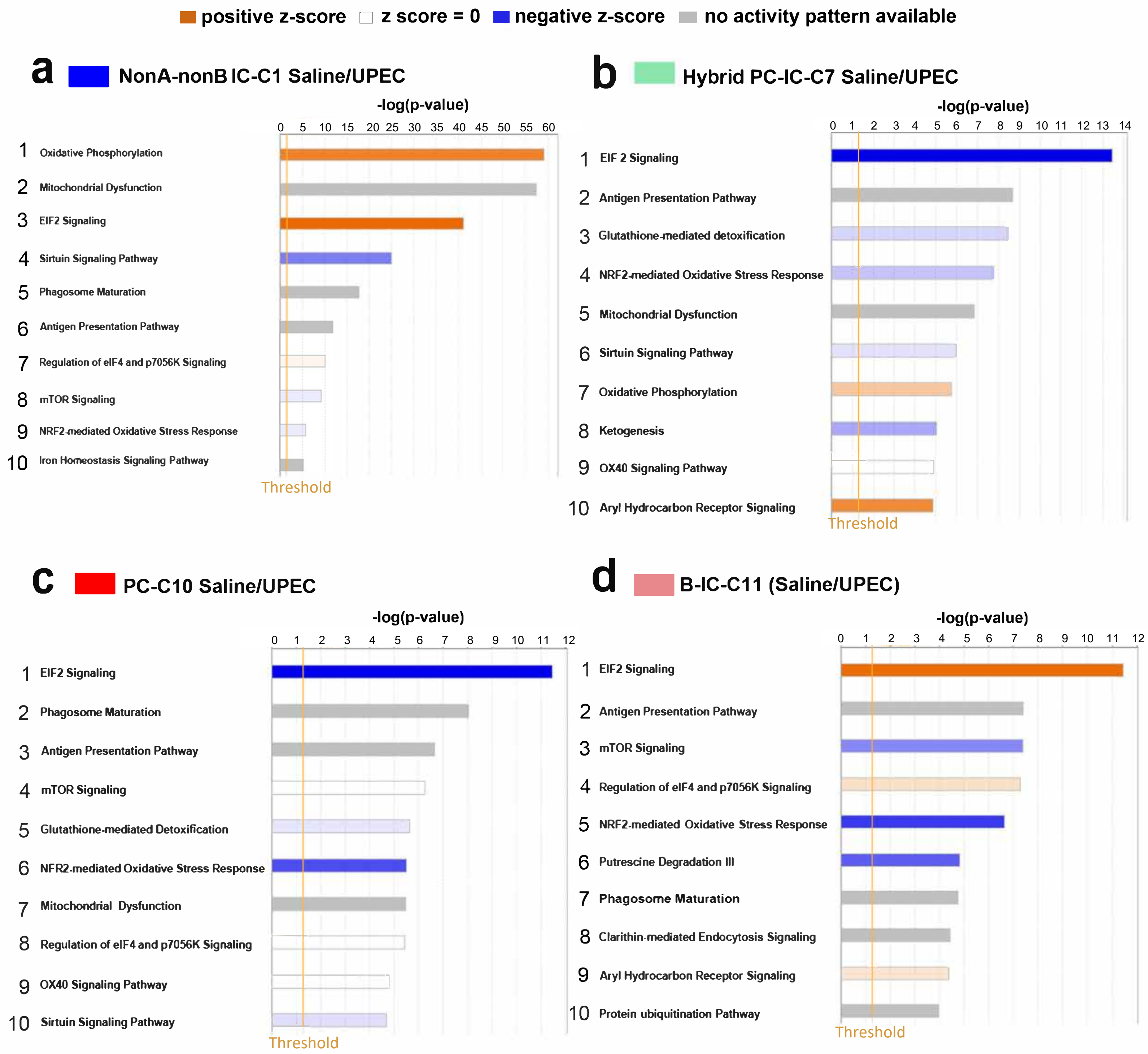

Ingenuity™ pathway analysis results for human collecting duct cell types without pathway changes in UPEC exposed compared to saline control exposed cells. Top 10 pathways in NonA-nonB ICs/cluster 1 (C1) (a), Hybrid PC-IC/cluster 7 (C7) (b), PCs/cluster 10 (C10) (c) and B-ICs/cluster 11 (d) did not have differences in the order of the top 10 biological pathways as ranked by -log(p-value). A threshold (orange vertical line) of less than 1.3 -log(p-value) was used to assign significance. Data were analyzed through the use of IPA (QIAGEN Inc., https://www.qiagenbioinformatics.com/products/ingenuity- pathway-analysis). IPA reports a p-value using a right-tailed Fisher's exact test without multiple comparisons. However, the IPA input data includes the log2 fold change and an adjusted p-value from the Seurat scRNAseq analysis.

Supplementary Figure 20: IC internalization and acidification of *E. coli* coated bioparticles in vitro

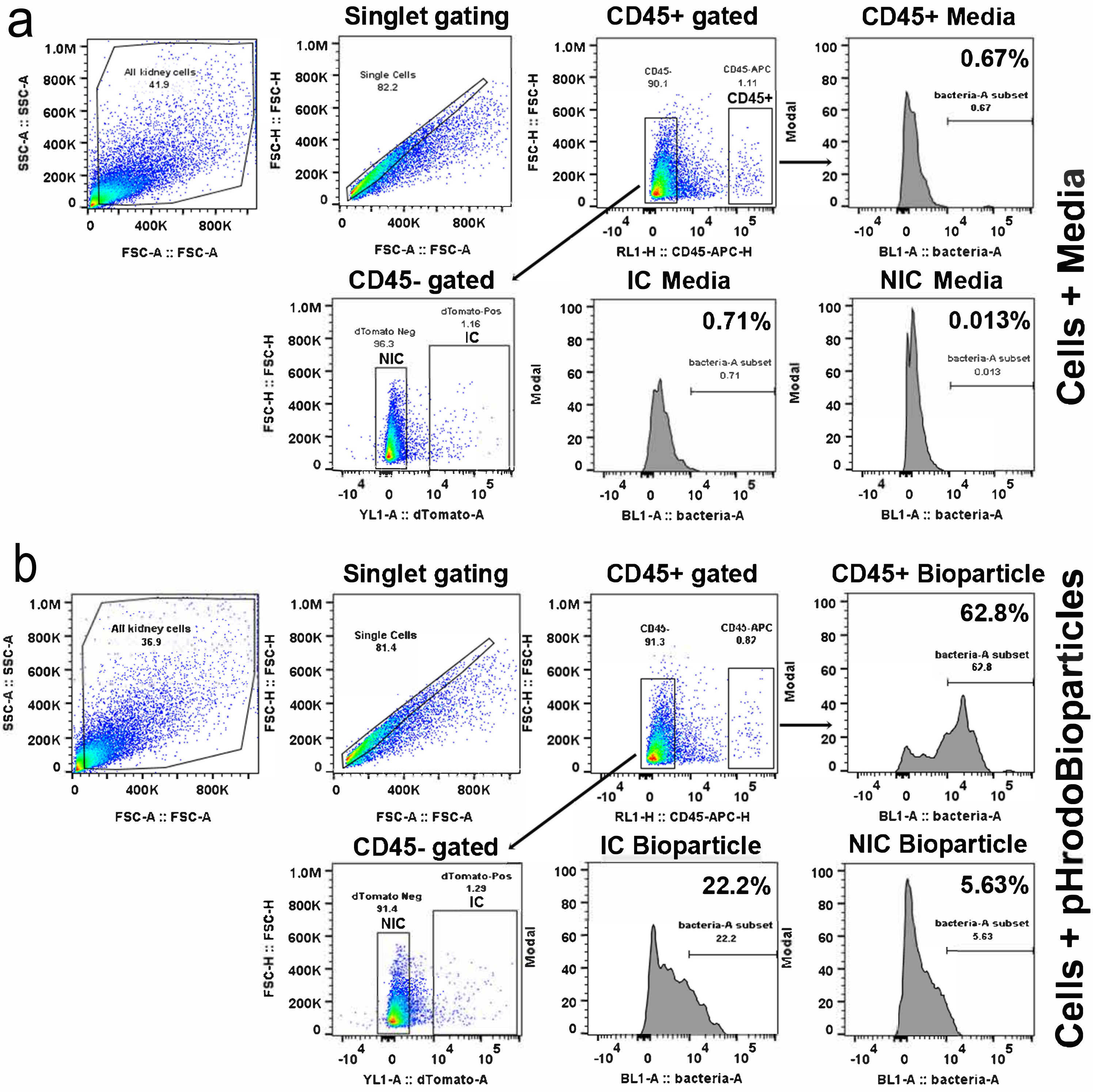

Murine kidney cell suspension from “IC-reporter” mice was prepared. Cells were analyzed after acquisition by flow cytometry into a CD45$^+$ fraction representing immune cells, red fluorescent protein variant tdTomato (tdT$^+$) fraction representing ICs and a CD45$^-$tdT$^-$ fraction representing the remainder of the kidney cells and exposed to media alone or media containing a pH sensitive pHrodo Green *E. coli* BioParticles that cause cells to exhibit increased green fluorescence if bioparticles are phagocytized and acid is secreted into the phagosome. (a) In cells exposed to media alone, background green fluorescence uptake by cells was 0.7%, 0.0% and 0.0% of the CD45$^+$ cells, tdT$^+$ ICs and tdT$^-$ non-IC kidney cells. (b) cells exposed to pHrodo Green *E. coli* BioParticles, 62.8% of CD45$^+$ cells, 22.2% of tdT$^+$ ICs and 5.6% tdT$^-$ non-IC kidney cells expressed green fluorescence. The increase in the proportion of green fluorescence positive cells was higher in tdT$^+$ ICs compared to tdT$^-$ non-IC kidney cells following exposure to *E. coli* coated bioparticles, p <10E-15. The increase in proportion of green fluorescence positive cells was significantly higher in CD45$^+$ cells than the tdT$^+$ ICs or tdT$^-$ non-IC kidney cells. Proportions were analyzed with a 2-tailed Fisher Exact test. The data was obtained from 4 mouse kidneys pooled from 2 mice and run in a single experiment. Source data are provided as a Source Data File.

Supplementary Figure 21: The gating strategy to enrich ICs from mice

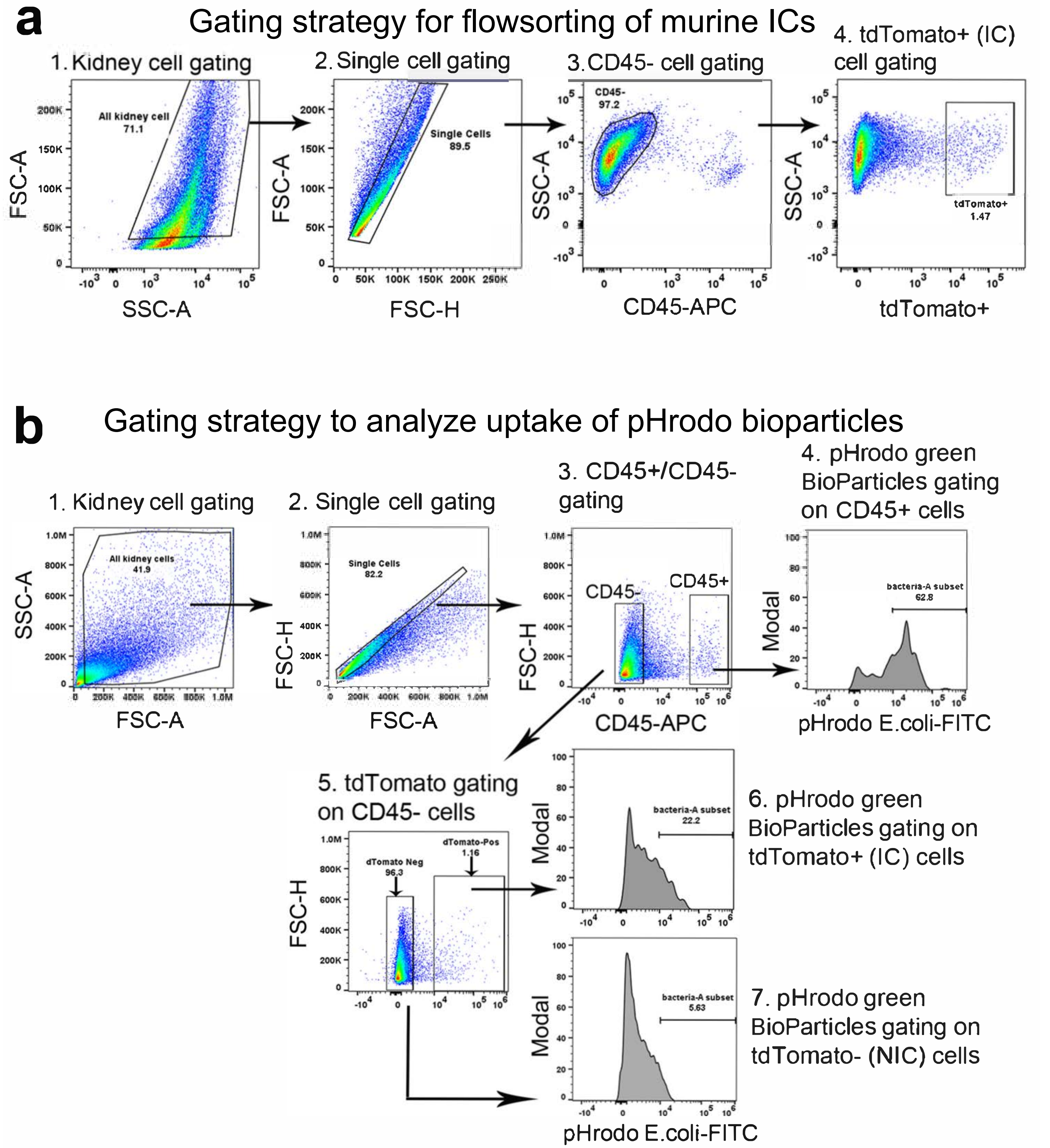

(a) The gating strategy to show the enrichment of murine kidney ICs from saline and UPEC treated mice by flowsorting from V-ATPase-cre+tdTomato+ (IC reporter) mice. CD45-tdTomato+ cells (IC) were collected and used to detect Atp6v1b1 mRNA expression reported in Figure 6g. (b) The gating strategy to analyze uptake of pHrodo Green *E. coli* BioParticles gating strategy to analyze pHrodo Green *E. coli* BioParticle uptake by CD45+, CD45-tdTomato+ (IC) and CD45-tdTomato- (NIC) cells. Kidney cell suspension from V-ATPase-cre+tdTomato+ (IC reporter) mice was analyzed for pHrodo Green *E. coli* BioParticle particle uptake in the gated cells. This strategy was used for Supplementary Figure 20.

Supplementary Table 1: Patient characteristics			
Tissue wt. (in gm)	Sex	Pathology Finding	Experimental Use
1.00	Male	Renal cell carcinoma	Targeted PCR for IC Enrichment
3.20	Female	Metanephric adenoma	Targeted PCR for IC Enrichment
1.06	Female	Endometrial carcinoma	Targeted PCR for IC Enrichment
1.19	Male	Focal segmental glomerulosclerosis	Targeted PCR for IC Enrichment
1.28	Male	Renal cell carcinoma	Single Cell RNAseq experiment
Participants were aged 36-77 years. Ethnicity was “European-American” for four patients and “other” for one patient.			

Supplementary Table 2: Genes used as cell type markers		
Renal cell type specific marker reported by Chen and colleagues		
Gene	Name	Cell type associated with
AGT	Angiotension	Proximal S3 cell
AQP2	Aquaporin 2	Principal cells
CALB1	Calbindin 1	Connecting tubule cells
LRP2	Megalin	Proximal tubule cells
SCNN1G	Sodium Channel Epithelial 1 Gamma Subunit	Principal cell
SLC4A1	Solute Carrier Family 4, Anion Exchanger, Member 1/ Band3	A-IC
SLC5A2	Solute Carrier Family 5 Member 2	Proximal S1 cell
SLC12A1	Solute Carrier Family 5 Member 2	Thick ascending limb cell
SLC14A2	Solute Carrier Family 14 Member 2	Descending thin limb cell
SLC22A6	Solute Carrier Family 22 Member 6	Proximal S3 cell
SLC26A4	Solute Carrier Family 26 Member 4/ Pendrin	B-IC
Conserved marker genes^ previously reported to be cell type markers		
ATP6V1G3	Vacuolar Proton Pump Subunit G 3	Intercalated cell marker
EHD3	EH Domain-Containing Protein 3	Glomerular endothelial cell
PLVAP	Plasmalemma Vesicle Associated Protein)	Vascular fenestrated endothelium
Conserved marker genes^ that differentiated one of the 12 clusters from others		
HSPA1A	Heat Shock Protein Family A (Hsp70) Member 1A	Differentiates cluster 3 from cluster 0
EGR1	Early Growth response 1	Differentiates clusters 3 and 0 from cluster 5
PRAP1	Proline Rich Acidic Protein 1	Cluster 2 specific marker
RBP4	Retinol Binding Protein 4	Cluster 6 specific marker
SLC8A1	Solute Carrier Family 8 Member A1/ Sodium/Calcium Exchanger 1	Cluster 7 specific marker
^ “top 9” conserved genes in clusters presented in Supplemental Tables 3-14		

Supplementary Table 3: Expression of key “phagosome maturation pathway” in A-IC, subtype A (cluster 0) relative to other clusters					
Gene Symbol	Gene name	Expression Log fold change	Adjusted p-value	Location of transcribed protein	Transcribed protein type
ATP6AP1	ATPase H+ transporting accessory protein 1	0.675	2.36E-41	cytoplasm	transporter
ATP6V0A4	ATPase H+ transporting V0 subunit a4	0.942	4.88E-53	cytoplasm	transporter
ATP6V0B	ATPase H+ transporting V0 subunit b	0.857	1.73E-43	cytoplasm	transporter
ATP6V0D2	ATPase H+ transporting V0 subunit d2	0.549	1.43E-24	cytoplasm	transporter
ATP6V0E1	ATPase H+ transporting V0 subunit e1	0.31	4.12E-10	cytoplasm	transporter
ATP6V1A	ATPase H+ transporting V1 subunit A	0.473	3.67E-30	plasma membrane	transporter
ATP6V1B1	ATPase H+ transporting V1 subunit B1	0.604	5.81E-30	cytoplasm	transporter
ATP6V1C2	ATPase H+ transporting V1 subunit C2	0.562	9.13E-36	cytoplasm	transporter
ATP6V1H	ATPase H+ transporting V1 subunit H	0.305	1.17E-18	cytoplasm	transporter
CTSC	cathepsin C	-0.448	1	cytoplasm	peptidase
CTSD	cathepsin D	0.829	2.27E-38	cytoplasm	peptidase
CTSH	cathepsin H	-0.384	1	cytoplasm	peptidase
DYNLL1	dynein light chain LC8-type 1	-0.35	1	cytoplasm	other
DYNLRB1	dynein light chain roadblock-type 1	-0.35	1	cytoplasm	other
HLA-A	major histocompatibility complex, class I, A	0.286	7.72E-11	plasma membrane	other
HLA-B	major histocompatibility complex, class I, B	0.294	7.03E-16	plasma membrane	transmembrane receptor
HLA-C	major histocompatibility complex, class I, C	0.40	2.19E-17	plasma membrane	other
HLA-DRA	major histocompatibility complex, class II, DR alpha	-1.742	1	plasma membrane	transmembrane receptor
HLA-DRB1	major histocompatibility complex, class II, DR beta 1	-1.903	1	plasma membrane	transmembrane receptor
HLA-E	major histocompatibility complex, class I, E	-0.474	0.0000937	plasma membrane	transmembrane receptor
PRDX1	peroxiredoxin 1	-0.391	1	cytoplasm	enzyme
TUBA1B	tubulin alpha 1b	-0.278	1	cytoplasm	other

Differential expression of representative “phagosome maturation” genes in Cluster 0 versus other clusters. The predicted phagosome maturation pathway involvement in human ICs included V-ATPase, cathepsin, cytoplasmic dynein, major histocompatibility complexes, tubulin and myeloperoxidase signaling. The scRNAseq fold changes and adjusted p-values calculated by Seurat are presented. Seurat uses the Wilcoxon rank sum test to generate a p-value for gene expression and a Bonferroni correction to account for a false discovery rate and calculate an adjusted p-value

Supplementary Table 4: Linear regression results for analysis of green fluorescent uptake by cells during intravital microscopy								
	Slope	R squared	Sy.x	F	DFn, DFd	P value	Deviation from zero?	values (n)
BT1-cell 0	23.75	0.51	110.00	121.40	1, 116	<1E-15	Significant	118
BT1-cell 1	23.35	0.47	115.90	108.90	1, 121	<1E-15	Significant	123
BT1-cell 2	-0.97	0.00	18.94	0.03	1, 19	0.854	Not Significant	21
BT1-cell 3	2.84	0.00	68.32	0.04	1, 21	0.843	Not Significant	23
BT1-cell 4	4.10	0.05	49.56	1.79	1, 32	0.190	Not Significant	34
BT1-cell 5	-11.74	0.06	49.58	1.53	1, 24	0.228	Not Significant	26
BT1-cell 6	18.29	0.33	122.30	59.95	1, 121	3.298E-12	Significant	123
BT1-cell 7	9.44	0.07	157.40	9.65	1, 121	0.002	Significant	123
BT1-cell 8	33.74	0.71	101.70	295.60	1, 121	<1E-15	Significant	123
BT1-cell 9	42.94	0.87	74.66	706.10	1, 110	<1E-15	Significant	112
BT1-cell 10	12.92	0.39	76.50	76.58	1, 121	1.5E-14	Significant	123
BT2-cell 0	26.94	0.34	171.20	58.45	1, 111	8.048E-12	Significant	113
BT2-cell 1	24.42	0.28	182.20	45.80	1, 117	5.499E-10	Significant	119
BT2-cell 2	18.12	0.28	135.80	45.96	1, 117	5.189E-10	Significant	119
BT2-cell 3	11.29	0.23	96.28	33.83	1,112	5.787E-08	Significant	123
BT2- cell 4	21.74	0.17	224.00	22.97	1,113	5.042E-06	Significant	123

Differential uptake of *E. coli* coated bioparticles by ICs in vivo. The linear regression results for each analyzed cell following intratubular injection of pHrodo Green *E. coli* BioParticles followed by intravital imaging and fluorescent quantification of GFP expression over is presented in table form. The slope representing the rate of increase in green fluorescence along with the R square , standard deviation of the values around the regression line (*Sy.X*), F distribution, degrees of freedom for the numerator and denominator (DFn and Dfd, p-values, whether the slopes differ significantly from a zero slope line and the number of values in each regression line are presented. Source data are provided as a Source Data File.

Supplementary Table 5: Primer sequences		
Mouse	Forward (5`-3`)	Reverse (5`-3`)
IC reporter mice (*Cre*)	CATTACCGGTCGATGCAACGAG	TGCCCCTGTTTCACTATCCAGG
Gapdh	CTGGAGAAACCTGCCAAGTA	TGTTGCTGTAGCCGTATTCA
Atp6v1b1	CCCTACGATTGAGCGGATCAT	TATATCCAGGAAAGCCACGGC
Human		
GAPDH	ACAGTTGCCATGTAGACC	TTTTTGGTTGAGCACAGG
ATP6V1B1	ATTTACAGGGGACATCCTAC	CCATTGATATCCAGAAAGTCC
SLC4A1	GAATTCTGGAAAAGATTCCCC	CACAAAGAGGAAGCGTATAG
SLC26A4	AGTTGATATTTGGTGGCTTG	TTGGTTGAAACATTGAGGAC
RNASE7	CTTTTTGTTGCTGTTTTCCC	CCCCCTTTCCATTTATTTCC

Supplementary Table 6. Key resources		
REAGENT or RESOURCE	SOURCE	IDENTIFIER
Antibodies		
Polyclonal Rabbit anti-Human c-KIT antibody	Dako Agilent, SantaClara, CA	Cat no. A4502
Polyclonal Chicken anti-Human V-type proton ATPase subunit E1	Sigma, Burlington, MA	Cat no. GW22284F
Monoclonal Mouse Anti-HSP 70	Santa Cruz Biotechnology, Dallas TX	Cat no. SC-32239
Monoclonal Rabbit Anti-EGR1	Cell Signaling, Danvers, MA	Cat no. 4153S
Polyclonal Rabbit Anti-SLC8A1	Sigma, Burlington, MA	Cat no. HPA070007
Polyclonal Goat Anti-AQP2	Santa Cruz Biotechnology TX	Cat no. SC-9882
Donkey Anti-Goat IgG Alexa Flour 488	Jackson Immuno, West Grove, PA	Cat no. 705545147
Donkey Anti-Chicken IgG Alexa Flour 488	Jackson Immuno, West Grove, PA	Cat no. 703545155
Donkey Anti-Rabbit IgG Alexa Flour 488	Jackson Immuno, West Grove, PA	Cat no. 711545152
Donkey Anti-Chicken Dylite 405	Jackson Immuno, West Grove, PA	Cat no. 703475155
Donkey Anti-Mouse Cy3	Jackson Immuno, West Grove, PA	Cat no. 715165150
Donkey Anti-Rabbit Cy3	Jackson Immuno, West Grove, PA	Cat no. 705165147
Donkey Anti-Chicken Cy3	Jackson Immuno, West Grove, PA	Cat no. 703165155
DAPI	BioLegend, San Diego, CA	Cat no. 422801
Bacterial and Virus Strains		
GFP expressing Uropathogenic *E. coli* strain CFT073	Mathew Mulvey, PhD, UniversityofUtah,SaltLake City, UT	
Biological Samples		
Human kidney samples	Cooperative Human Tissue Network Mid-western Division, Ohio State, Columbus OH	www.CHTN.org
Deposited Data		
https://hpcwebapps.cit.nih.gov/ESBL/Database/IU-Data/Human-c-Kit-Sorted-Single- Cell-RNASeq.htm	WebpageHostedbyEpithelial Systems Biology lab, NHLBI, Bethesda, MD	
https://www.ncbi.nlm.nih.gov/geo/query/acc.cgi?acc=GSE159805	National Center for Biotechnology Information gene expression ombnibus (GEO)	Accession number: GSE159805
Experimental Models: Organisms/Strains		
V-ATPase B1-Cre transgenic mice (C57BL/6 background)	Raoul Nelson, MD, University of Utah, Salt Lake City, UT	
tdTomato-lo*xp* homozygous mice [B6.Cg-Gt(ROSA)26Sor$^{tm9(CAG\text{-}tdtomato)Hze}$/J	Jackson Laboratories, Bar Harbor, ME	Stock no.007909
Software and Algorithms		
Keyence BZ Analyzer	Keyence, Osaka, Japan	
Leica Application Suite X	Leica, Wetzlar, Germany	
Single cell analysis software	CellRanger 2.1.0, 10x Genomics, Pleasanton, CA	http://support.10xgenomics.com/)
Ingenuity™ CanonicalPathwayAnalysis	Ingenuity Systems, Redwood City, CA	http://www.ingenuitypathway.org
Imaris microscope image analysis software	Oxford Instruments, Zurich,Switzerland	https://imaris.oxinst.com
Other		
pHrodo Green *E. coli* BioParticles™	Molecular probes, Invitrogen, Carlsbad, CA	Cat no. P35381
Human tissue protein expression data	Human Protein Atlas	http://www.proteinatlas.org
Kidney cell transcriptomics data	Kidney Cell Explorer	https://cello.shinyapps.io/kidneycellexplorer/
