# Supplementary material for: Kidney intercalated cells are phagocytic and acidify internalized uropathogenic Escherichia coli
Source: Nat Commun. 2021 Apr 23;12:2405. doi: 10.1038/s41467-021-22672-5 (PMC8065053; doi:10.1038/s41467-021-22672-5)
Supplement: Supplementary file 3 — Description of Additional Supplementary Files [file 41467_2021_22672_MOESM3_ESM.pdf]

Description of Additional Supplementary Files

File Name: Supplementary Software 1
Description: This document outlines the code use to generate the RNA velocity data presented in Figure 5.
