# Supplementary material for: Kidney intercalated cells are phagocytic and acidify internalized uropathogenic Escherichia coli
Source: Nat Commun. 2021 Apr 23;12:2405. doi: 10.1038/s41467-021-22672-5 (PMC8065053; doi:10.1038/s41467-021-22672-5)
Supplement: Supplementary file 4 — Supplementary Software 1 [file 41467_2021_22672_MOESM4_ESM.pdf]

Supplementary Software 1: RNA Velocity Script.R

```
library(velocyto.R)
library(pagoda2)
library(stringr)
#Wont run full script but will run when i is set to each and full code copy pasted
for(i in c("CKIT_Saline","CKIT_UPEC")){
newclusters<-read.csv(paste0("/mnt/neph/Jered/schwaderer/CKIT_clusters.csv"))
rename<-newclusters[grep(i,newclusters[,1]),2]
names(rename)<-
str_extract(pattern="(?<=_)[ACGT]+",string=newclusters[grep(i,newclusters[,1]),1])
ldat <- read.loom.matrices(paste0("/mnt/neph/Jered/schwaderer/",i,".loom"))
emat <- ldat$spliced
# this dataset has already been pre-filtered, but this is where one woudl do some filtering
emat <- emat[,colSums(emat)>=1e3]
emat<-emat[!duplicated(rownames(emat)),]
colnames(emat)<-str_extract(pattern="(?<=:)[ACGT]+",string=colnames(emat))
 emat<-emat[,intersect(colnames(emat),names(rename))]
r <- Pagoda2$new(emat,modelType='plain',trim=10,log.scale=T)
pdf(paste0("/mnt/neph/Jered/schwaderer/",i,"-VarianceFit.pdf"),width=10,height=10)
r$adjustVariance(plot=T,do.par=T,gam.k=10)
dev.off()
r$calculatePcaReduction(nPcs=100,n.odgenes=3e3,maxit=300)
r$makeKnnGraph(k=30,type='PCA',center=T,distance='cosine');
r$getKnnClusters(method=multilevel.community,type='PCA',name='multilevel')
r$getEmbedding(type='PCA',embeddingType='tSNE',perplexity=50,verbose=T)
pdf(paste0("/mnt/neph/Jered/schwaderer/",i,"-Auto_tSNE.pdf"),width=10,height=10)
r$plotEmbedding(type='PCA',embeddingType='tSNE',show.legend=F,mark.clusters=T,min.grou
p.size=10,shuffle.colors=F,mark.cluster.cex=1,alpha=0.3,main=paste0('cell clusters LPS',i,'hr'))
dev.off()
pcaTop<-rownames(r$misc$PCA$v[1:20,])
```

```
write.csv(pcaTop,paste0("/mnt/neph/Jered/schwaderer/",i,"Auto_Top20PC1Genes.csv"))

r$clusters$PCA[[1]]<-rename[names(r$clusters$PCA[[1]])]

#pdf(paste0("/mnt/neph/Jered/scLPS/RNAvelocity/renamedClusters/LPS",i,"hr-
tSNE_relabeled2.pdf"),width=10,height=10)

#r$plotEmbedding(type='PCA',embeddingType='tSNE',show.legend=F,mark.clusters=T,min.gro
up.size=10,shuffle.colors=F,mark.cluster.cex=1,alpha=0.3,main=paste0('cell clusters LPS',i,'hr'))

#dev.off()

emat <- ldat$spliced; nmat <- ldat$unspliced

colnames(emat)<-str_extract(pattern="(?<=:)[ACGT]+",string=colnames(emat))

emat<-emat[,intersect(colnames(emat),names(rename))]

colnames(nmat)<-str_extract(pattern="(?<=:)[ACGT]+",string=colnames(nmat))

nmat<-nmat[,intersect(colnames(nmat),names(rename))]

emat <- emat[,rownames(r$counts)]; nmat <- nmat[,rownames(r$counts)]; # restrict to cells that
passed p2 filter

# take cluster labels

cluster.label <- r$clusters$PCA[[1]]

#cluster.label <- rename

cell.colors <- pagoda2:::fac2col(cluster.label)

# take embedding

emb <- r$embeddings$PCA$tSNE

#emb <-read.csv("/mnt/neph/Jered/schwaderer/CKIT_UMAP.csv",row.names=1)

emb <-readRDS("/mnt/neph/Jered/schwaderer/CKIT_UMAP.rds")

emb<-emb[grep(i,rownames(emb)),]

rownames(emb)<-str_extract(pattern="(?<=_)[ACGT]+",string=rownames(emb))

 #rownames(emb)

emb<-emb[colnames(emat),]

pcaOverWrite<-read.csv(paste0("/mnt/neph/Jered/schwaderer/CKIT_PCAs.csv"),row.names=1)

pcaOverWrite<-pcaOverWrite[grep(i,rownames(pcaOverWrite)),]

rownames(pcaOverWrite)<-
str_extract(pattern="(?<=_)[ACGT]+",string=rownames(pcaOverWrite))
```

```
pcaOverWrite<-t(pcaOverWrite)[,colnames(t(r$reductions$PCA))]

cell.dist <- as.dist(1-armaCor(pcaOverWrite))

emat <- filter.genes.by.cluster.expression(emat,cluster.label,min.max.cluster.average = 0.5)

nmat <- filter.genes.by.cluster.expression(nmat,cluster.label,min.max.cluster.average = 0.05)

length(intersect(rownames(emat),rownames(emat)))

fit.quantile <- 0.02

emat<-emat[,intersect(colnames(emat),labels(cell.dist))]

rvel.cd <-
gene.relative.velocity.estimates(emat,nmat,deltaT=1,kCells=20,cell.dist=cell.dist,fit.quantile=fit.q
uantile)

#emb2<-data.frame(emb)

#emb2$cell.colors<-as.character(cell.colors)

#emb2$cluster.label<-as.numeric(cluster.label)

#write.csv(emb2,paste0("/mnt/neph/Jered/scLPS/RNAvelocity/renamedClusters/LPS",i,"hr-
TSNEdata.csv"))


pdf(paste0("/mnt/neph/Jered/schwaderer/",i,"_UMAP_Overlay_VelocityVectors.pdf"),width=10,h
eight=10)

show.velocity.on.embedding.cor(emb,rvel.cd,n=300,scale='sqrt',cell.colors=ac(cell.colors,alpha=
0.5),cex=0.8,arrow.scale=5,show.grid.flow=TRUE,min.grid.cell.mass=0.5,grid.n=40,arrow.lwd=1
,do.par=F,cell.border.alpha = 0.1)

dev.off()

}
```
