# Supplementary material for: Kidney intercalated cells are phagocytic and acidify internalized uropathogenic Escherichia coli
Source: Nat Commun. 2021 Apr 23;12:2405. doi: 10.1038/s41467-021-22672-5 (PMC8065053; doi:10.1038/s41467-021-22672-5)
Supplement: Supplementary file 5 — Reporting Summary [file 41467_2021_22672_MOESM5_ESM.pdf]

nature research

Corresponding author(s): Andrew Schwaderer
Vijay Saxena

Last updated by author(s): 2020-22-12

Reporting Summary

Nature Research wishes to improve the reproducibility of the work that we publish. This form provides structure for consistency and transparency in reporting. For further information on Nature Research policies, see our Editorial Policies and the Editorial Policy Checklist.

Statistics

For all statistical analyses, confirm that the following items are present in the figure legend, table legend, main text, or Methods section.

n/a	Confirmed	
☐	☒	The exact sample size (*n*) for each experimental group/condition, given as a discrete number and unit of measurement
☐	☒	A statement on whether measurements were taken from distinct samples or whether the same sample was measured repeatedly
☐	☒	The statistical test(s) used AND whether they are one- or two-sided<br>*Only common tests should be described solely by name; describe more complex techniques in the Methods section.*
☐	☒	A description of all covariates tested
☐	☒	A description of any assumptions or corrections, such as tests of normality and adjustment for multiple comparisons
☐	☒	A full description of the statistical parameters including central tendency (e.g. means) or other basic estimates (e.g. regression coefficient) AND variation (e.g. standard deviation) or associated estimates of uncertainty (e.g. confidence intervals)
☐	☒	For null hypothesis testing, the test statistic (e.g. *F*, *t*, *r*) with confidence intervals, effect sizes, degrees of freedom and *P* value noted<br>*Give P values as exact values whenever suitable.*
☒	☐	For Bayesian analysis, information on the choice of priors and Markov chain Monte Carlo settings
☒	☐	For hierarchical and complex designs, identification of the appropriate level for tests and full reporting of outcomes
☐	☒	Estimates of effect sizes (e.g. Cohen's *d*, Pearson's *r*), indicating how they were calculated

Our web collection on statistics for biologists contains articles on many of the points above.

Software and code

Policy information about availability of computer code

Data collection	No code was used for data collection
Data analysis	Cell Ranger 3.0.2, Seurat v3, Ingenuity Canonical Pathway Analysis (April 2019 release version), Velocyto CLI v0.17, Imaris software(x64v7.2.1), Graph pad Prism (v8 and v9), FlowJo version 10 software, Microsoft PowerPoint 16, Adobe Photoshop CS5.1, Keyence BZ II analyzer and Leica Application Suite X. Human Protein Atlas (https://www.proteinatlas.org/), Kidney cell explorer (https://cello.shinyapps.io/kidneycellexplorer/)

For manuscripts utilizing custom algorithms or software that are central to the research but not yet described in published literature, software must be made available to editors and reviewers. We strongly encourage code deposition in a community repository (e.g. GitHub). See the Nature Research guidelines for submitting code & software for further information.

Data

Policy information about availability of data

All manuscripts must include a data availability statement. This statement should provide the following information, where applicable:

- Accession codes, unique identifiers, or web links for publicly available datasets
- A list of figures that have associated raw data
- A description of any restrictions on data availability

scRNAseq processed data can be found at: https://hpcwebapps.cit.nih.gov/ESBL/Database/IU-Data/Human-c-Kit-Sorted-Single-Cell-RNASeq.htm
scRNAseq raw data is available at NCBI gene expression omnibus (GEO). Its GEO accession number is GSE159805 (https://www.ncbi.nlm.nih.gov/geo/query/acc.cgi?acc=GSE159805).
scRNAseq integrated Seurat cluster analysis script can be found at Github repository with this link:
https://github.com/visaxena2870/Schwaderer_scRNAseq_Seurat_IntegratedAnalysis.R/blob/main/Schwaderer_scRNAseq_Seurat_IntegratedAnalysis.R

The source immunohistochemistry images obtained from the Human Protein Atlas (http://www.proteinatlas.org) for supplemental material 6A-6C may be found at;
https://www.proteinatlas.org/ENSG00000120738-EGR1/tissue/kidney#img (A)
https://www.proteinatlas.org/ENSG00000204389-HSPA1A/tissue/kidney#img (B)
https://www.proteinatlas.org/ENSG00000183023-SLC8A1/tissue/kidney#img (C)
The https://cello.shinyapps.io/kidneycellexplorer/ website was used to compare our scRNAseq findings to comparable past murine studies. For findings not related to the aforementioned links we have submitted source data with this manuscript. Additionally source data can be obtained by contacting the lead author.

Field-specific reporting

Please select the one below that is the best fit for your research. If you are not sure, read the appropriate sections before making your selection.

- [x] Life sciences
- [ ] Behavioural & social sciences
- [ ] Ecological, evolutionary & environmental sciences

For a reference copy of the document with all sections, see nature.com/documents/nr-reporting-summary-flat.pdf

Life sciences study design

All studies must disclose on these points even when the disclosure is negative.

Sample size	ScRNAseq is powerful tool to access gene and cell type in hundreds of cells at a time but cost prohibitive. Human single cell RNAseq was performed on 1861 cells, In vivo mRNA expression on flowsorted IC was performed on 11-12 "IC reporter" mice which was found sufficient to reveal statistical significance. 10 mice were used to obtain 2 successful tubule microperfusions for bacteria and 2 for Bioparticles. Additionally one control mouse was used (n = 11 total mice used). The number of animals was not determined initially due to the pilot nature of this study. Results and data were obtained until the research team concluded that sufficient information was available to show reproducibility and sufficient tubules and cells for Imaris analysis. For PCR studies to evaluate human ICs gene expression (e.g. Cell type marker and RNASE7), we used 4 tissue sample to show individual to individual similarities and variabilities.
Data exclusions	No data was excluded from study
Replication	Because our scRNAseq data was obtained from obtained from a single patient we replicated our key finding using immmunoflourescence (IF) demonstrating that human hybrid PC-IC cells and A-IC subtypes marker expression was consistent between individuals. IF/PCR was performed on multiple individual samples with all results shown for PCR and representative images for IF. For intravital studies we confirmed UPEC uptake and Bioparticle uptake in multiple mice. Bioparticle experiment was consistent with in vitro flowcytometry experiment. Some attempt of replication of intravital experiment were unsuccessful because we were unable to cannulate the tubular lumen or needle was clogged with bacteria or Bioparticles (number are listed in methodology).
Randomization	For single cell analysis we randomly selected kidney cell suspension to be exposed to saline and UPEC. After 1 hr exposure, cells were collected and processed for sequencing.
Blinding	For the intravital experiments we did not perform blinding because it was obvious when bacterial or bioparticles were injected because of their GFP expression (as opposed to control). We did not blind for the scRNAseq because a dedicated core processed the samples and analyzed the results using a unbiased approach/standard methodology.

Reporting for specific materials, systems and methods

We require information from authors about some types of materials, experimental systems and methods used in many studies. Here, indicate whether each material, system or method listed is relevant to your study. If you are not sure if a list item applies to your research, read the appropriate section before selecting a response.

Materials & experimental systems

n/a	Involved in the study	
[ ]	[x]	Antibodies
[x]	[ ]	Eukaryotic cell lines
[x]	[ ]	Palaeontology and archaeology
[ ]	[x]	Animals and other organisms
[ ]	[x]	Human research participants
[x]	[ ]	Clinical data
[x]	[ ]	Dual use research of concern

Methods

n/a	Involved in the study	
[ ]	[x]	ChIP-seq
[ ]	[x]	Flow cytometry
[x]	[ ]	MRI-based neuroimaging

Antibodies

Antibodies used	Dead cells were removed from human kidney tissue using dead cell removal microbeads (Miltenyi Biotec, Cat No. 130-090-101), microbead for Human CD45 (Miltenyi Biotec, Cat. no 30-045-801) and C-KIT cells (Miltenyi Biotec, Cat. no. 130-091-332) were used for enrichment of human ICs (Miltenyi Biotec). Anti-mouse CD45-APC (eBioscience, Cat. no. 17-0451), pHrodo green E.coli Bioparticles (Invitrogen, Cat. no. P35381), ATP6V1E1 antibody produced in chicken (Sigma, Cat. no. GW22284), rabbit anti-human CKIT (Cat. no. A450229-2, Dako Agilent). All secondary antibody (anti-Chicken Cy3, anti-Rabbit AF488) were purchased from Jackson

Immunoresearch. Monoclonal Mouse Anti-HSP 70 (Santa Cruz, Cat no. SC-32239), Monoclonal Mouse Anti-HSP70 , Monoclonal Rabbit Anti-EGR1 (Cell Signaling Cat no. 4153S) and Polyclonal Goat Anti-AQP2 (Santa Cruz, Cat no. SC-9882)

Validation

Antibodies used and validation resource:
Mouse CD45-APC ThermoFihser website (91 references including PMID: 29755322 on ThermoFisher Website)
SLC8A1:https://www.proteinatlas.org/ENSG00000183023-SLC8A1/antibody (sample HPA070007)
HSPA1A (HSP70):https://www.proteinatlas.org/ENSG00000204389-HSPA1A/antibody sample CAB032815)
EGR1:https://www.proteinatlas.org/ENSG00000120738-EGR1/antibody (patient)CAB019427
KIT:https://www.proteinatlas.org/ENSG00000157404-KIT/antibody CAB003288
VATPASE E1:PMID: 31166705
AQP2: PMID: 31166705
Mouse CD45-APC used for flowcytometry has been shown to stain C57BL/6 splenocytes on Biolegend website and cited by several investigators.
SLC8A1:https://www.proteinatlas.org/ENSG00000183023-SLC8A1/antibody (sample HPA070007)
HSPA1A (HSP70):https://www.proteinatlas.org/ENSG00000204389-HSPA1A/antibody sample CAB032815)
EGR1:https://www.proteinatlas.org/ENSG00000120738-EGR1/antibody (patient)CAB019427
KIT:https://www.proteinatlas.org/ENSG00000157404-KIT/antibody CAB003288
VATPASE E1:PMID: 31166705
AQP2: PMID: 31166705

Animals and other organisms

Policy information about studies involving animals; ARRIVE guidelines recommended for reporting animal research

Laboratory animals

tdTomato-flox /flox were purchased from Jackson laboratory and crossed with ATP6v1b1-Cre mice (Kindly provided by Dr Raoul Nelson, Utah) to produce "IC reporter mice". Male "IC-reporter" mice were used for live intravital imaging and Female mice were used for induction of UTI. For IC flowsorting and intravital experiments mice ages ranged from 55-103 days.

Wild animals

No wild animals were used in the study.

Field-collected samples

No field collected samples were used in the study.

Ethics oversight

Murine studies were approved by the Institutional Animal Care and Use Committee (IACUC) at the Indiana University School of Medicine by the protocol number 11333

Note that full information on the approval of the study protocol must also be provided in the manuscript.

Human research participants

Policy information about studies involving human research participants

Population characteristics

Supplemental material S3 outlines the clinical characteristics from the patients that the kidney margins were obtained from Cooperative Human Tissue Network (Columbus, OH, www.CHTN.org). Fresh human kidney biopsy samples from the Cooperative Human Tissue Network (Columbus, OH, www.CHTN.org). Kidney tissue was obtained from the normal margins of adjacent kidney of patients undergoing renal surgeries were cut into small, ~2-4 mm pieces, placed in sterile Dulbecco's Modified Eagle Medium (DMEM) and shipped overnight in cold packs.

Recruitment

No patient recruitment was done by the study team. the samples were obtained from the Cooperative Human Tissue Network.

Ethics oversight

Human research was exempt per Indiana University Institutional Review Board protocol 1802253259

Note that full information on the approval of the study protocol must also be provided in the manuscript.

ChIP-seq

Data deposition

- [x] Confirm that both raw and final processed data have been deposited in a public database such as GEO.
- [ ] Confirm that you have deposited or provided access to graph files (e.g. BED files) for the called peaks.

Data access links
May remain private before publication.

Processed Data Link: can be found at:
https://hpcwebapps.cit.nih.gov/ESBL/Database/IU-Data/Human-c-Kit-Sorted-Single-Cell-RNASeq.htm

Files in database submission

Raw data deposited in NCBI GEO can be found at:

https://www.ncbi.nlm.nih.gov/geo/query/acc.cgi?acc=GSE159805

Genome browser session (e.g. UCSC)

https://genome.ucsc.edu

Methodology

Replicates

scRNAseq was performed on a human kidney sample that was divided into saline vs UPEC exposed fractions for analysis

Sequencing depth

CellRanger 3.0.2 was utilized to process the raw sequence data generated. Briefly, CellRanger uses bcl2fastq to demultiplex raw base sequence calls generated from the sequencer into sample-specific FASTQ files. The FASTQ files were then aligned to the reference genome with RNA-seq aligner STAR. The aligned reads were traced back to the individual cells and the gene expression level of individual genes were quantified based on the number of UMIs (unique molecular indices) detected in each cell.

The filtered gene-cell barcode matrices generated with CellRanger were used for further analysis with the R package Seurat development version 3.0.0.9000 58, 59. Quality control (QC) of the data was implemented as the first step in our analysis. We first filtered out genes that detected in less than five cells and cells with less than 200 genes. To further exclude low-quality cells in downstream analysis we used the function Outlier from R package scater together with visual inspection of the distributions of number of genes, UMIs, and mitochondrial gene content 60. Cells with extremely high or low number of detected genes/UMIs were excluded.

Saline: Estimated Number of Cells 484, Fraction Reads in Cells 69.3%Mean Reads per Cell 1,040,791Median Genes per Cell 2,710Total Genes Detected 19,000Median UMI Counts per Cell 8,366

UPEC: Estimated Number of Cells 434 Fraction Reads in Cells 74.6%Mean Reads per Cell 1,185,924 Median Genes per Cell 2,721 Total Genes Detected 18,532 Median UMI Counts per Cell 8,660

Antibodies

No antibodies were used for scRNAseq Experiment.

Peak calling parameters

To integrate the single cell data from the treated and untreated samples, functions "FindIntegrationAnchors" and "IntegrateData" from Seurat v3 were implemented with dimensionality of 30. The integrated data was then scaled and PCA was performed. Clusters were then identified with the Seurat functions "FindNeighbors" and "FindClusters" using a resolution of 0.8 and the first 12 PCs. The "FindConservedMarkers" function was used to identify cell cluster marker genes. To compare average gene expression within the same cluster between cells of different samples, "AverageExpression" function was applied. "FindMarkers" function was also used to investigate the differences of gene expression induced by treatment within the same cell cluster. Biological relevance of identified gene was evaluated using Ingenuity™ canonical Pathway Analysis (April 2019 version). The cell clusters were visualized using the t-Distributed Stochastic Neighbor Embedding (t-SNE) plots and Uniform Manifold Approximation and Projection (UMAP) plots. R packages ggplot2 (Wickham, 2016) and ggrepel (https://github.com/slowkow/ggrepel) were used to plot the average gene expression. Violin plots (VlnPlot) and feature plots (FeaturePlot) were used to visualize specific gene expressions across clusters and different sample conditions.

Data quality

Quality control (QC) of the data was implemented as the first step in our analysis. We first filtered out genes that detected in less than five cells and cells with less than 200 genes. To further exclude low-quality cells in downstream analysis we used the function Outlier from R package scater together with visual inspection of the distributions of number of genes, UMIs, and mitochondrial gene content 60. Cells with extremely high or low number of detected genes/UMIs were excluded. In addition, cells with greater than 25% mitochondrial reads were also filtered out. After removing likely multiplets and low-quality cells, the gene expression levels for each cell were normalized with the NormalizeData function in Seurat.

Software

CellRanger 3.0.2 was utilized to process the raw sequence data generated. Briefly, CellRanger uses bcl2fastq to demultiplex raw base sequence calls generated from the sequencer into sample-specific FASTQ files. The FASTQ files were then aligned to the reference genome with RNA-seq aligner STAR. The aligned reads were traced back to the individual cells and the gene expression level of individual genes were quantified based on the number of UMIs (unique molecular indices) detected in each cell. For RNA Velocity analysis velocyto v0.17 pipeline was used.

Flow Cytometry

Plots

Confirm that:

- [x] The axis labels state the marker and fluorochrome used (e.g. CD4-FITC).
- [x] The axis scales are clearly visible. Include numbers along axes only for bottom left plot of group (a 'group' is an analysis of identical markers).
- [x] All plots are contour plots with outliers or pseudocolor plots.
- [x] A numerical value for number of cells or percentage (with statistics) is provided.

Methodology

Sample preparation	For in vitro phagocytosis assay, murine renal cells from IC-reporter mice were prepared by gentlemacs dissociator (Miltenyi Biotec) and accumax enzymatic digestion solution (Innvovative cell Tech). Dead cells were removed by dead cell removal microbead (Miltenyi). Cells were filtered throuh 70 micron bucket filter and red cells were lysed (Biolegend). Celle were surface labeled with CD45-APC for 30 min at 4 degree C. E.coli pHrodo bioparicles were then added for 15 min at 37 degree C. Particle uptake was stopped by incubating cells on ice and immediately acquired on flowcytometer. For In vivo V-ATPase mRNA expression, IC-reporter mice were challenged with UPEC strain CFT073 for 1 hr and then renal IC were flowsorted from renal cell suspension after gating out doublet and CD45+ cells. Tdtomato+ cells were sorted for mRNA analysis.
Instrument	Attune flowcytometer (Invitrogen) was used for phagocytosis assay. FACAria (Becton Dickinson) was used for flowsorting of IC cells.
Software	Flowjo version 10
Cell population abundance	tdTomato+ cells (presumed IC) in IC-reporter mice kidney ranged from 0.5-1.5% of the total population after gating out CD45 + cells prior to sort. After sort it became 90-95% in enriched fraction. Purity was determined by analyzing a vial of cells for expression of tdT fluorescence prior to sorting and cells after sorting on flowcytometer with average 90% purity. IC purity was confirmed by RT-PCR of marker gene mRNA expression on the sorted cells compared to Non-IC and CD45+ cells as described earlier (PubMed: 28468965)
Gating strategy	Murine IC Flowsorting: Renal cell suspension was first analyzed by size (FSC/SSC) gating on flowcytometer. Cells very close to the bottom left were excluded (debris) and rest were gated ,in the next window cells were gated for doublets exclusion, in the next window doublet neg cells were gated for CD45-APC, then CD45- cells were gated, finally tdT+ cells (IC) were visulaized and collected.<br>Phagocytosis Assay: Cell suspension from IC reporter mice was prepared and exposed to Bioparticles and analyzed on flowcytometer. After gating out doublets, CD45-tdt+, tCD45-dT- and CD45+ cells were analyzed for FITC-pHrodo Bioparticle uptake using Flowjo V10 software. ICs were defined as CD45-tdT+, Non-IC were CD45-tdT- cell population compared to renal immune cells which were CD45+ alone.

☒ Tick this box to confirm that a figure exemplifying the gating strategy is provided in the Supplementary Information.
