# Supplementary material for: Kidney intercalated cells are phagocytic and acidify internalized uropathogenic Escherichia coli
Source: Nat Commun. 2021 Apr 23;12:2405. doi: 10.1038/s41467-021-22672-5 (PMC8065053; doi:10.1038/s41467-021-22672-5)

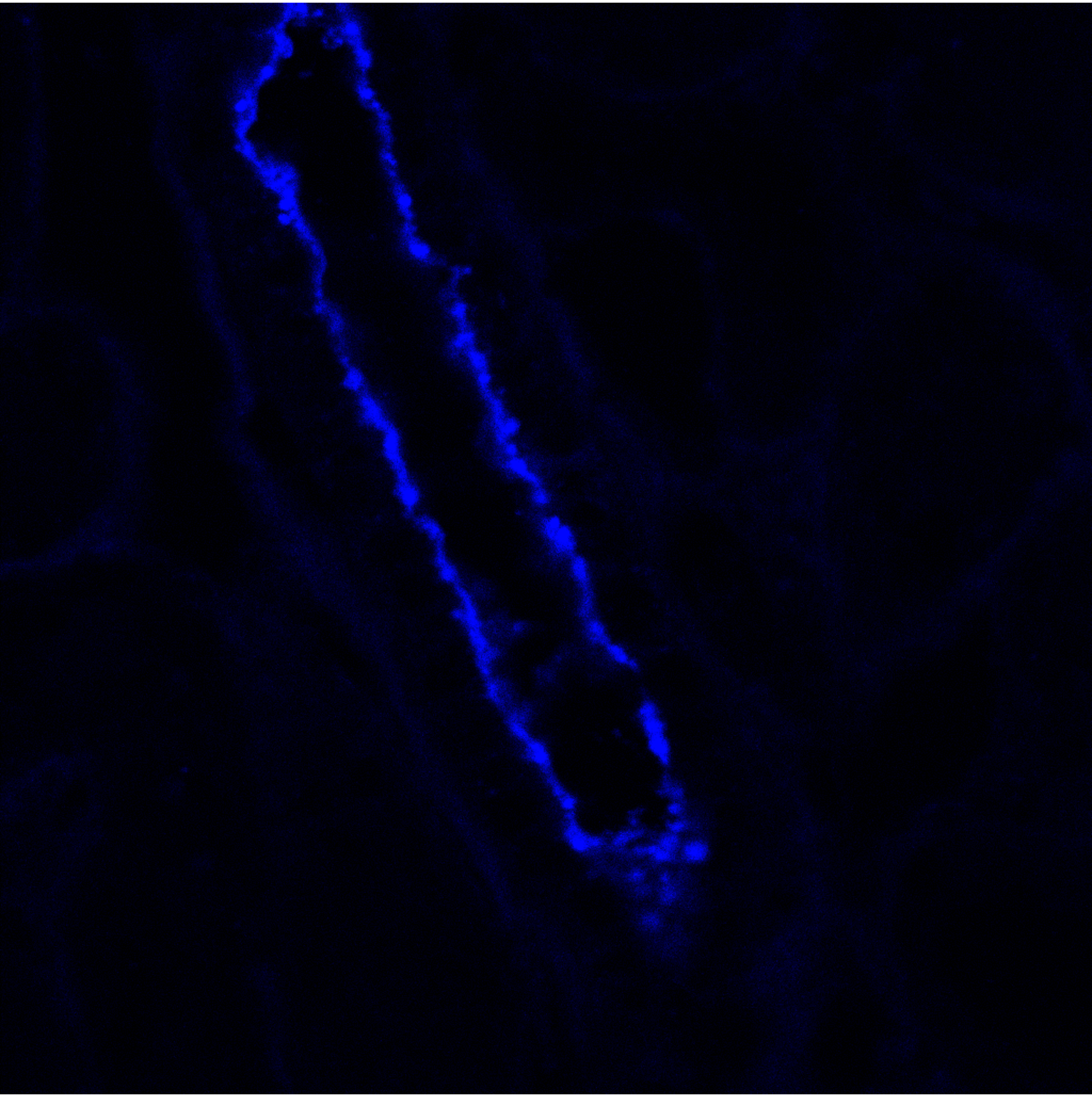

Supplement: Supplementary file 6 — Source Data [file 41467_2021_22672_MOESM6_ESM.zip › source data/Figure 3 source data/Figure-3a-blue-AQP2-human kidney.pdf]
