# Supplementary material for: Kidney intercalated cells are phagocytic and acidify internalized uropathogenic Escherichia coli
Source: Nat Commun. 2021 Apr 23;12:2405. doi: 10.1038/s41467-021-22672-5 (PMC8065053; doi:10.1038/s41467-021-22672-5)
Supplement: Supplementary file 6 — Source Data [file 41467_2021_22672_MOESM6_ESM.zip › source data/figure 7 source data/replicate mouse-not shown in manuscript-Intravital injection of green UPEC-red ICs without uptake (bottom left) and with uptake ( yellow ICs-top)-.pdf]

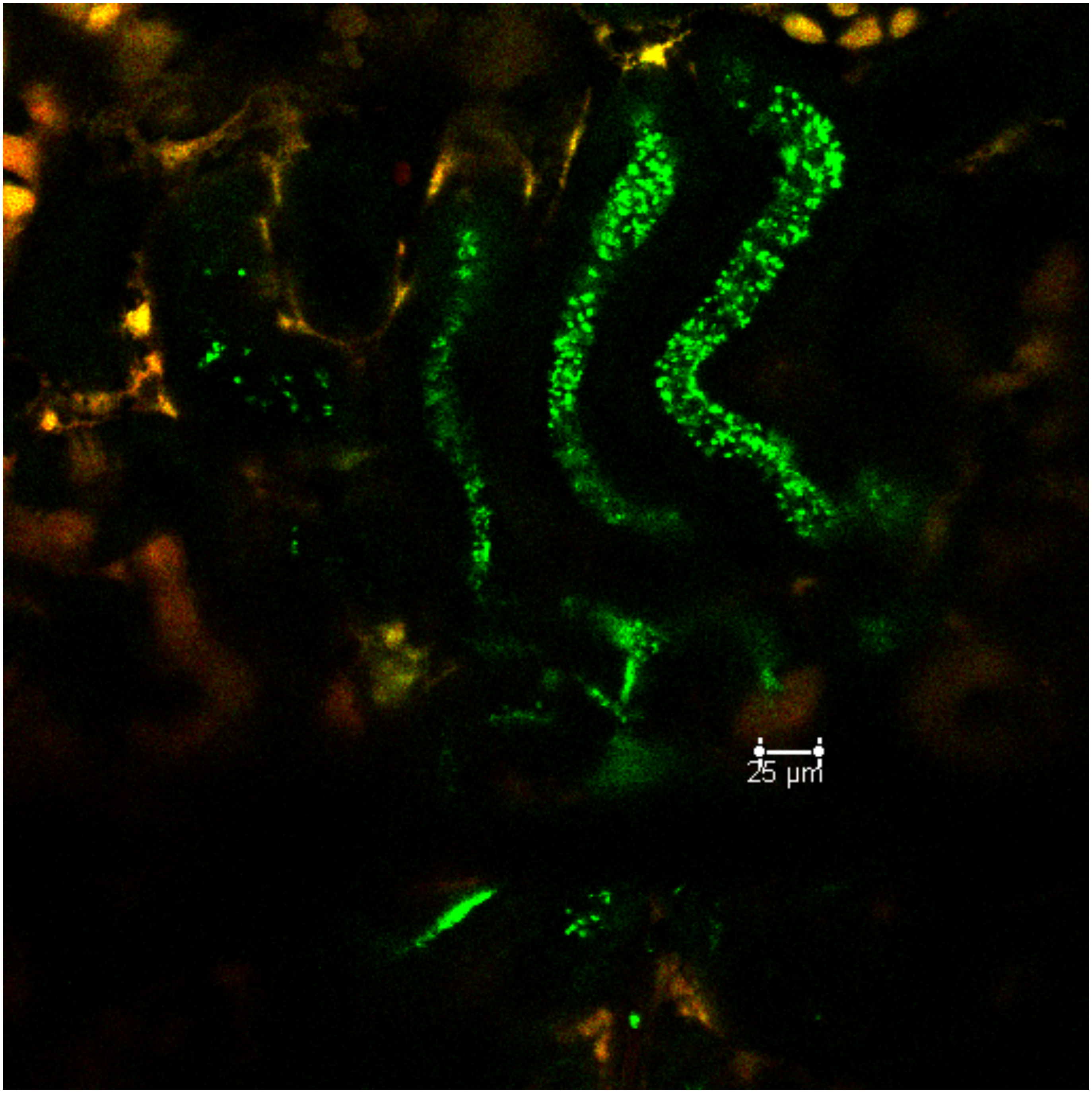
25 μm
