# Supplementary material for: Kidney intercalated cells are phagocytic and acidify internalized uropathogenic Escherichia coli
Source: Nat Commun. 2021 Apr 23;12:2405. doi: 10.1038/s41467-021-22672-5 (PMC8065053; doi:10.1038/s41467-021-22672-5)

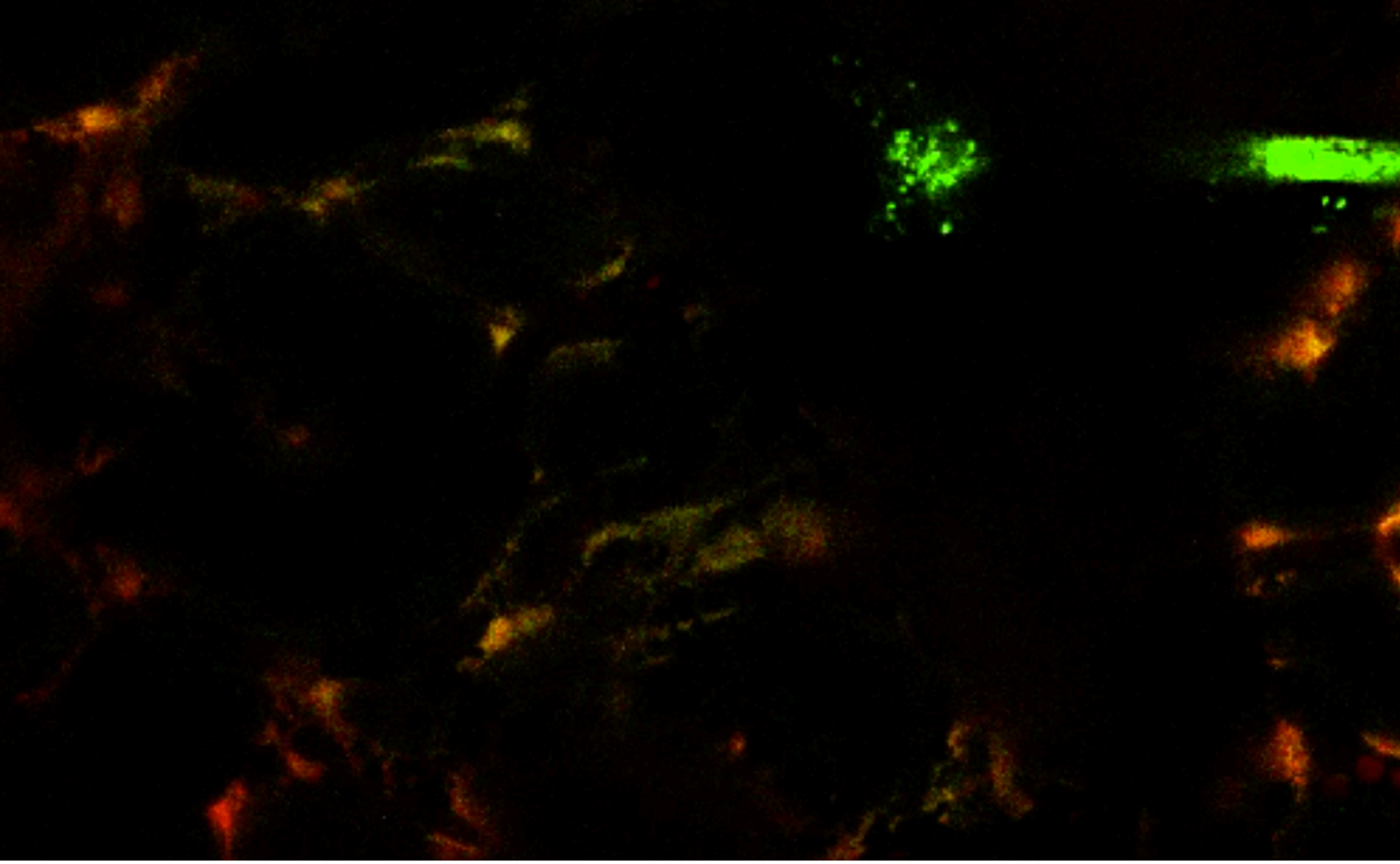

Supplement: Supplementary file 6 — Source Data [file 41467_2021_22672_MOESM6_ESM.zip › source data/Figure 8 source data/replicate mouse-not shown in manuscript - bioparticle (green) infusion into ICs (red) ~14 min.pdf]
